# PBP-A, a cyanobacterial dd-peptidase with high specificity for amidated muropeptides, exhibits pH-dependent promiscuous activity harmful to *Escherichia coli*

**DOI:** 10.1038/s41598-024-64806-x

**Published:** 2024-06-18

**Authors:** Gol Mohammad Dorrazehi, Matthias Winkle, Martin Desmet, Vincent Stroobant, Gamze Tanriver, Hervé Degand, Damien Evrard, Benoît Desguin, Pierre Morsomme, Jacob Biboy, Joe Gray, Karolina Mitusińska, Artur Góra, Waldemar Vollmer, Patrice Soumillion

**Affiliations:** 1 Louvain Institute of Biomolecular Science and Technology, UCLouvain, Place Croix du Sud 4-5, 1348 Louvain-la-Neuve, Belgium; 2 Ludwig Institute for Cancer Research, Brussels, Belgium; 3 de Duve Institute, UCLouvain, Brussels, Belgium; 4 Centre for Bacterial Cell Biology, Biosciences Institute, Newcastle University, Richardson Road, Newcastle upon Tyne, NE2 4AX UK; 5 Biosciences Institute, Newcastle University, Richardson Road, Newcastle upon Tyne, NE2 4AX UK; 6 Tunneling Group, Biotechnology Centre, Silesian University of Technology, 44-100 Gliwice, Poland; 7 Present Address: Benchmark Animal Health Ltd, 1 Pioneer Building, Edinburgh Technopole, Milton Bridge, Penicuik, EH26 0GB UK; 8 Present Address: Department of Biochemistry, University of Cambridge, 80 Tennis Court Road, Cambridge, CB2 1GA UK

**Keywords:** Penicillin-binding proteins, Penicillin-binding proteins, Synthetic biology

## Abstract

Penicillin binding proteins (PBPs) are involved in biosynthesis, remodeling and recycling of peptidoglycan (PG) in bacteria. PBP-A from *Thermosynechococcus elongatus* belongs to a cyanobacterial family of enzymes sharing close structural and phylogenetic proximity to class A β-lactamases. With the long-term aim of converting PBP-A into a β-lactamase by directed evolution, we simulated what may happen when an organism like *Escherichia coli* acquires such a new PBP and observed growth defect associated with the enzyme activity. To further explore the molecular origins of this harmful effect, we decided to characterize deeper the activity of PBP-A both in vitro and in vivo. We found that PBP-A is an enzyme endowed with dd-carboxypeptidase and dd-endopeptidase activities, featuring high specificity towards muropeptides amidated on the d-iso-glutamyl residue. We also show that a low promiscuous activity on non-amidated peptidoglycan deteriorates *E. coli’s* envelope, which is much higher under acidic conditions where substrate discrimination is mitigated. Besides expanding our knowledge of the biochemical activity of PBP-A, this work also highlights that promiscuity may depend on environmental conditions and how it may hinder rather than promote enzyme evolution in nature or in the laboratory.

## Introduction

**Figure 1:**
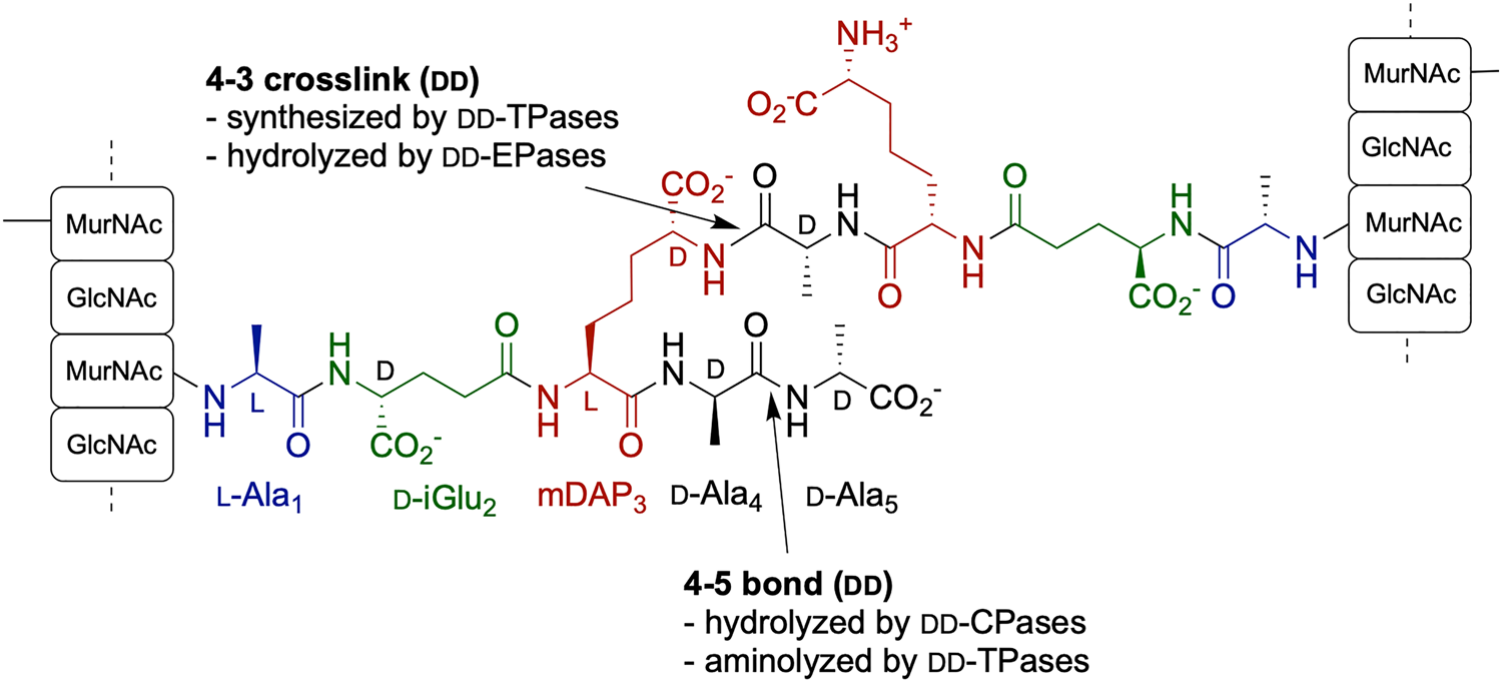
Chemical structure of bridged stem peptides in the peptidoglycan. The mDAP_3_ side chain of the acceptor peptide (bottom) is bridged to the D-Ala_4_ residue of the donor peptide (top). The acceptor peptide is represented in its precursor pentapeptide state although d-Ala_5_ is generally removed upon PG maturation by d-carboxypeptidases or dd-transpeptidases. The 4–3 crosslink is synthesized by dd-transpeptidases and can be hydrolyzed by DD-endopeptidases.

Bacterial peptidoglycan (PG) is an essential scaffold surrounding the plasma membrane that maintains the cell shape and integrity^1–3^. Gram-negative bacteria have an outer membrane (OM) and a thin, mesh-like PG layer that resides in the periplasm. The unique PG structure is composed of glycan strands made of alternating *N*-acetylglucosamine (Glc*N*Ac) and *N*-acetylmuramic acid (Mur*N*Ac), which are connected via short, cross-linked peptide stems attached to the Mur*N*Ac residues^4,5^. In *Escherichia coli*, the stem peptide is initially synthesized as l-Ala-d-iGlu-mDAP-d-Ala-d-Ala pentapeptide, which carries a meso-diaminopimelic acid (mDAP or m-A_2_pm) at position 3 with a main chain α-carbon in the l-configuration and a side chain α-carbon in the d-configuration^4,6^. Peptides are cross-linked via the carboxyl group of d-Ala at position 4 and the ε-amino group of the mDAP at position 3 (Fig. 1). These so called 4–3 crosslinks are biosynthesized by d-alanyl-d-alanine transpeptidase enzymes (DD-TPases) recognizing d-Ala-d-Ala motif as substrate and cleaving the terminal d-Ala during the transpeptidation. In some species, alternative 3–3 bridging between the main and side chains of two mDAP residues is catalyzed by structurally unrelated ld-TPases that will release the d-Ala in position 4^7^. Besides being crosslinked, muropeptides are also trimmed by various carboxypeptidases (dd-, ld- and dl-CPases) and cleaved by endopeptidases (dd- and ld-EPases) and amidases, while glycan chains are cleaved by lytic transglycosylases (LT)^3,8^.

dd-TPase, dd-EPase and dd-CPase enzymes belong to the same family of d-Alanyl-d-Alanine-peptidases (dd-peptidases), which are often called penicillin binding proteins or PBPs because they are covalently inhibited by β-lactam antibiotics such as penicillins^9,10^. Indeed, β-lactam antibiotics share structural similarities with d-Alanyl-d-Alanine substrate and react with dd-peptidases to form stable acyl-enzymes, leading to their inhibition and bacterial lysis^11^. On the other hand, serine β-lactamases (SBLs) are members of the same superfamily of penicillin recognizing proteins but have evolved as hydrolases of β-lactam antibiotics as a resistance mechanism. Since the PG and its biosynthetic enzymes are probably more ancient than the inhibitory penicillins which are themselves probably more ancient than their inactivating enzymes, it is generally accepted that serine β-lactamases evolved from ancestral PBPs. This evolutionary relationship is supported by a conserved acylation mechanism by β-lactams and, despite a barely detectable sequence similarity, a conserved structural fold^12–15^. To date, the highest (~ 30%) sequence identity is found between a family of cyanobacterial low molecular weight PBPs and class A SBLs^15,16^. These PBPs are found only in cyanobacteria and do not belong to well characterized PBP classes. They were named PBP-A because of their phylogenetic proximity to class A SBLs. The enzyme from *Thermosynechococcus elongatus* has been characterized and, based on its hydrolytic activity on surrogate thioester substrates, is considered as a d-Alanyl-d-Alanine-carboxypeptidase (dd-CPase) enzyme^16,17^ even though its biological function has not been investigated.

The interconversion of phylogenetically related enzymes catalyzing different reactions is an important goal in protein engineering because it should provide insight into the molecular determinants underlying the exquisite catalytic properties of enzymes and their evolutionary emergence. When compared to PBP-A, a key difference in class A β-lactamases is the presence of a catalytic glutamate in the omega loop flanking the active site and involved in activating the hydrolytic water molecule in the deacylation of the penicilloyl-enzyme. Guided by the structure of PBP-A, the introduction of a glutamate in the structurally aligned position afforded an enzyme with a 90-fold increase in β-lactamase activity. However, this activity is still five orders of magnitude lower compared to a natural β-lactamase and the variant is unable to confer any penicillin resistance to its *E. coli* expression host^17^. Multiple subsequent attempts to convert PBP-A into a phenotypically active β-lactamase through rational design and directed evolution failed (unpublished results). Hence, the simplistic view of a PBP evolving by acquiring a few mutations that would progressively increase its β-lactamase activity is probably far from reality, and it raises fundamental questions on evolutionary trajectories leading to the emergence of new catalytic activities. In this specific case, we have notably accumulated clues that harmful effects, related to PBP-A expression in *E. coli*, may constitute an important obstacle for its evolutionary conversion.

In this work, we characterize the activity of PBP-A with small synthetic peptides and purified muropeptides as substrates. We also evaluated the implication of PBP-A activity in deleterious effects associated with its expression in the periplasm of *E. coli* and characterized the phenotypes of strains under various growth conditions. Altogether, our study reveals the biochemical properties of PBP-A and emphasizes the significant impact on *E. coli* when it acquires this enzyme, alongside the inherent challenges associated with its directed evolution.

## Results

Wild type PBP-A presents an extra N-terminal domain (residues 1–92) predicted as a transmembrane anchor followed by a proline-rich spacer. We have previously shown that the soluble PBP domain can be expressed without this N-terminal domain in the periplasm of *Escherichia coli*, thus facilitating its purification and biochemical characterization in vitro^16,17^. All the results presented here are obtained with this soluble domain fused to a C-terminal His-tag, that we called PBP-A for the ease of reading.

### PBP-A is a dd-carboxypeptidase with high specificity for d-iGln-containing peptides

**Figure 2:**
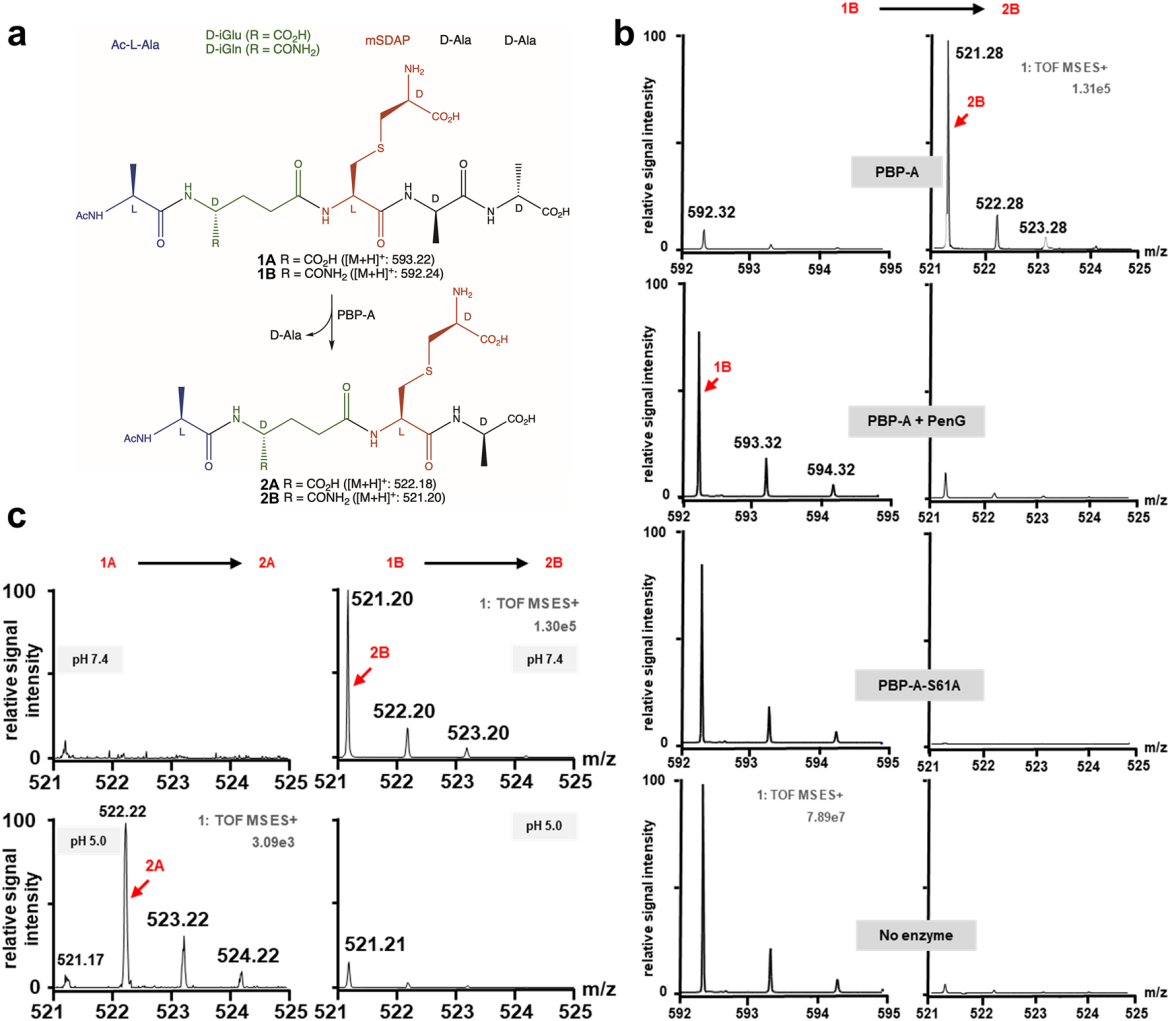
Activity assays of PBP-A on synthetic peptides. (**a**) dd-carboxypeptidase activity was assayed with two synthetic pentapeptides **1A** and **1B** featuring, from N- to C-ter, *N*-acetylated l-Ala (blue)**,** free or amidated d-iso-Glu (d-iGlu or d-iGln; green), mimic of mDAP (mSDAP, red) and C-terminal d-Ala-d-Ala (black). Stereochemistry of mSDAP is identical to that of mDAP; a sulfur (S) replaces the methylene (CH_2_) in gamma position of the side chain. Hydrolysis of C-terminal d-Ala generates respective tetrapeptides **2A** and **2B**. (**b**) Hydrolytic activity of PBP-A on amidated pentapeptide at pH 7.0. The spectra represent detection of substrate **1B** (left) and product **2B** (right) in assays of PBP-A with or without preincubation with penicillin G covalent inhibitor, catalytic mutant (S61A) of PBP-A and a control reaction without enzyme. (**c**) Detection of amidated product **2B** and non-amidated product **2A** in assay of PBP-A (~ 1.3 µM) incubated 1 h with pentapeptides (2 mM) **1A** and **1B,** respectively, at pH 5.0 and 7.4. Relative signal intensity is normalized to the absolute number of ion counts observed for the maximal intensity of product **2A** at pH 5.0 (bottom left spectrum) or product **2B** at pH 7.4 (top right spectrum) for left or right spectra respectively. In panels (**b** and **c**), the values indicated below “1: TOF MS ES+ ” represents the ion current observed for the peak of maximal intensity. Vertically, intensity scales are identical for each spectra within a column. Presented data are representative of multiple experiments.

To get better insight into the biochemical properties of PBP-A, the purified enzyme was initially incubated with synthetic surrogate pentapeptide substrates mimicking natural muropeptides (Fig. 2a). Since incorporating a meso-diaminopimelic acid residue in chemical synthesis does not allow a proper stereochemical control, a l-cysteine residue was introduced in position 3 and the thiol was subsequently modified with a d-chloroalanine. The obtained mimic of mDAP (called mSDAP) positions the l- and d-centers at the respective main and side chains and differs from mDAP only by a sulfur instead of a methylene in the gamma position. Additionally, the synthetic pentapeptides comprise an acetylated N-terminal l-Ala, an amidated or non-amidated d-iso-glutamyl residue in position 2 (d-iGlu and d-iGln, respectively) and a C-terminal d-Ala-d-Ala group. Glutamate amidation is a known peptidoglycan modification in some bacterial species^5,18,19^.

After incubation of enzyme (~ 1.3 µM) with both pentapeptides (2 mM) for 1 h, the products were analyzed by liquid chromatography–mass spectrometry (LC–MS). As shown in Fig. 2b we easily observed the tetrapeptide hydrolytic product (**2B**) when the amidated substrate (**1B**) was used at neutral pH. The starting substrate is almost completely hydrolyzed and, as control experiments, the activity is not observed either if the enzyme is absent, or if a catalytic mutant is used or if PBP-A is preincubated with penicillin. Tripeptides and crosslinked peptides indicative of respective ld-carboxypeptidase and DD-transpeptidase activities were not observed. Using time-dependent capillary electrophoresis analysis, we estimated the turnover rate in the range of 10^−2^ s^−1^ at 2 mM peptide concentration (Supplementary Fig. S1). Interestingly, we could not detect any activity with the non-amidated substrate (**1A**) indicating a strong discrimination against d-iGlu in position 2 (Fig. 2c, top left). Since a protonated carboxylic acid is more similar to a carboxamide than a negatively charged carboxylate, we evaluated the activity of PBP-A with the synthetic peptides at a more acidic pH. As shown in the bottom of Fig. 2c, lowering the pH to 5 results in a significant decreased activity on amidated substrate but also allowed the detection of carboxypeptidase activity on the non-amidated peptide, indicating that, indeed, substrate discrimination is reduced upon protonation. Note that MS spectra are displayed with a 40-fold increased sensitivity for the detection of **2A** compared to **2B** product. Hence, PBP-A activity at pH 5.0 is still higher on amidated **1B** substrate when compared to **1A**.

### PBP-A shows promiscuous dd-carboxypeptidase and dd-endopeptidase activities on d-iGlu-containing peptidoglycan

**Figure 3:**
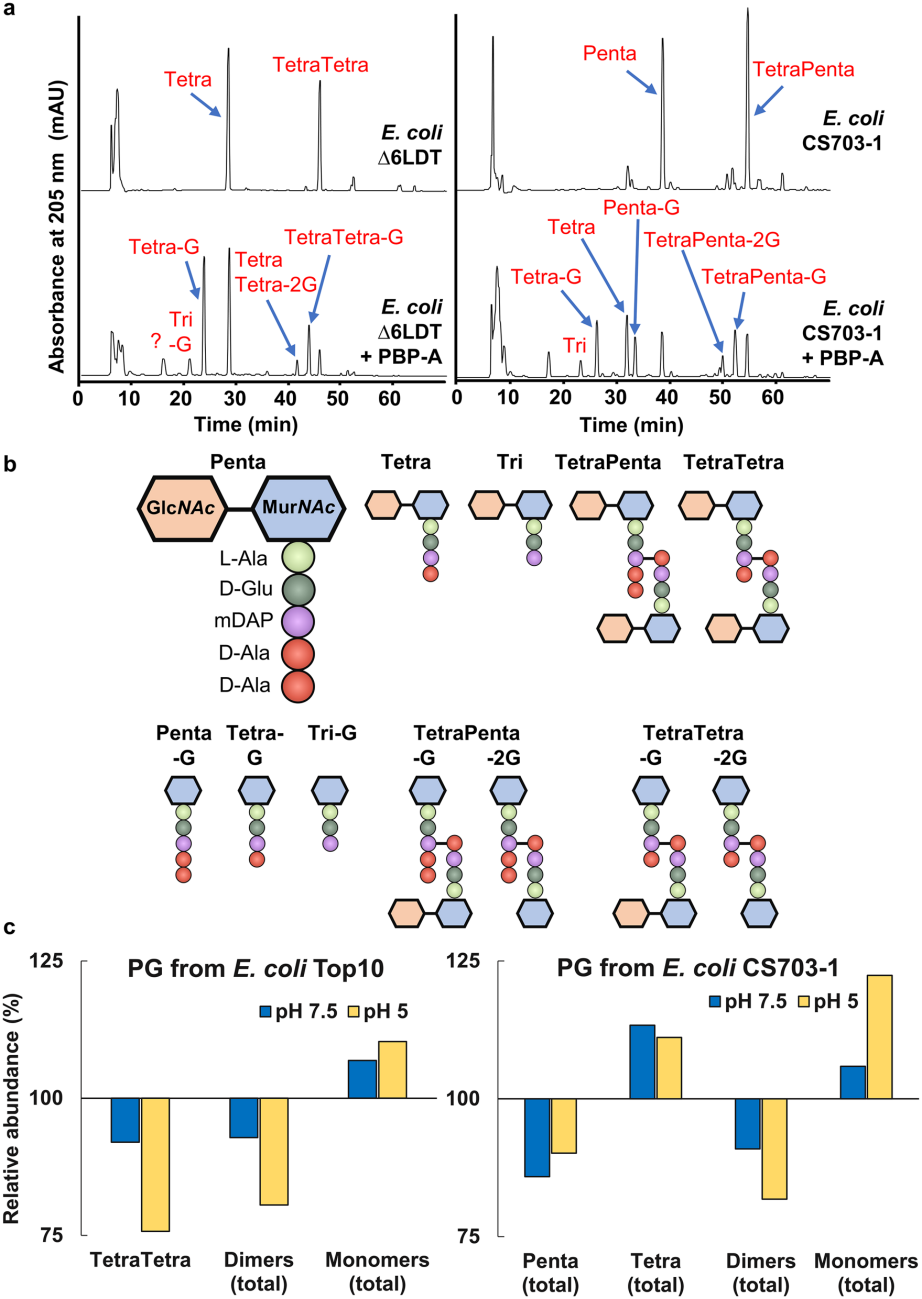
Activity of PBP-A on purified peptidoglycan (PG) from various *E. coli* strains. PG samples were incubated with PBP-A and treated with cellosyl prior to HPLC–MS analysis. (**a**) dd-EPase activity at pH 7.5 is highlighted with PG from BW25113∆6LDT (rich in tetrapeptides). Calculated abundances of all muropeptides by UV absorbance of this assay are available in Supplementary Tables S1 and S2. Additional HPLC chromatograms and MS–MS analyses for detected peptides are available in Supplementary Fig. S2. (**b**) Schematic representation of different observed fragments. (**c**) Quantitative analysis of different peptides detected in assays of PBP-A with PG of *E. coli* TOP10 and CS703-1 strains, relative to the control (no PBP-A treatment) samples in assays under pH 7.5 and 5.0. Abundance of all muropeptides detected at different pH in both assays are available in Supplementary Tables S2 and S3. Presented data are representative of multiple experiments.

We then evaluated the activity of PBP-A on the PG of *E. coli* which is known not to be amidated^20^. PBP-A was assayed with peptidoglycan (PG) extracts from three *E. coli* strains: TOP10, BW25113∆6LDT strain, which lacks all ld-transpeptidases^21^ and, therefore, is homogeneous with abundant tetrapeptides, and CS703-1 strain, which lacks five PBPs^22^ and thus is rich in pentapeptides. After incubation of PBP-A with PG extracts at pH 7.5 or 5, samples were digested with cellosyl lysozyme (muramidase) and the released muropeptides were analyzed by HPLC–MS. Representative chromatograms with main identified peaks as well as proposed activities and quantitative analysis for the assays are presented in Fig. 3, Supplementary Figs. S2, S3 and Supplementary Tables S1 to S3. At both pH, we observed some peptidase activity on *E. coli*’s PG while the catalytic mutant PBP-A-S61A didn’t show any activity (Supplementary Fig. S3). Results indicate that PBP-A is endowed with both dd-CPase and dd-EPase activities. Indeed, significant increase in amount of Tetra muropeptide at the expense of Penta muropeptide (see Fig. 3 for naming) is indicative of cleavage between the two C-terminal d-Alanine (dd-CPase activity) while increase in total monomers at the expense of dimers indicates cleavage of 4–3 crosslinks (dd-EPase activity). At acidic pH, the dd-EPase activity of PBP-A is at least two-fold higher compared to neutral pH (Fig. 3c). However, this increased activity is not observed for the carboxypeptidase activity in apparent contradiction with the pH effect observed with synthetic peptides.

Unexpectedly, Fig. 3a also show the detection of tripeptides that may result from ld-CPase activity on tetrapeptides or ld-dipeptidase activity on pentapeptides, as well as the detection of Glc*NAc* removal from Glc*NAc*-Mur*NAc*-peptides. These activities most likely originate from contaminating endogenous enzymes, as they were observed when PBP-A was purified from a periplasmic expression system in *E. coli* but disappeared when the enzyme was purified using a cytoplasmic expression system (see Supplementary Fig. S3).

### PBP-A activity in the periplasm of *E. coli* is generating a pH-dependent fitness cost

**Figure 4:**
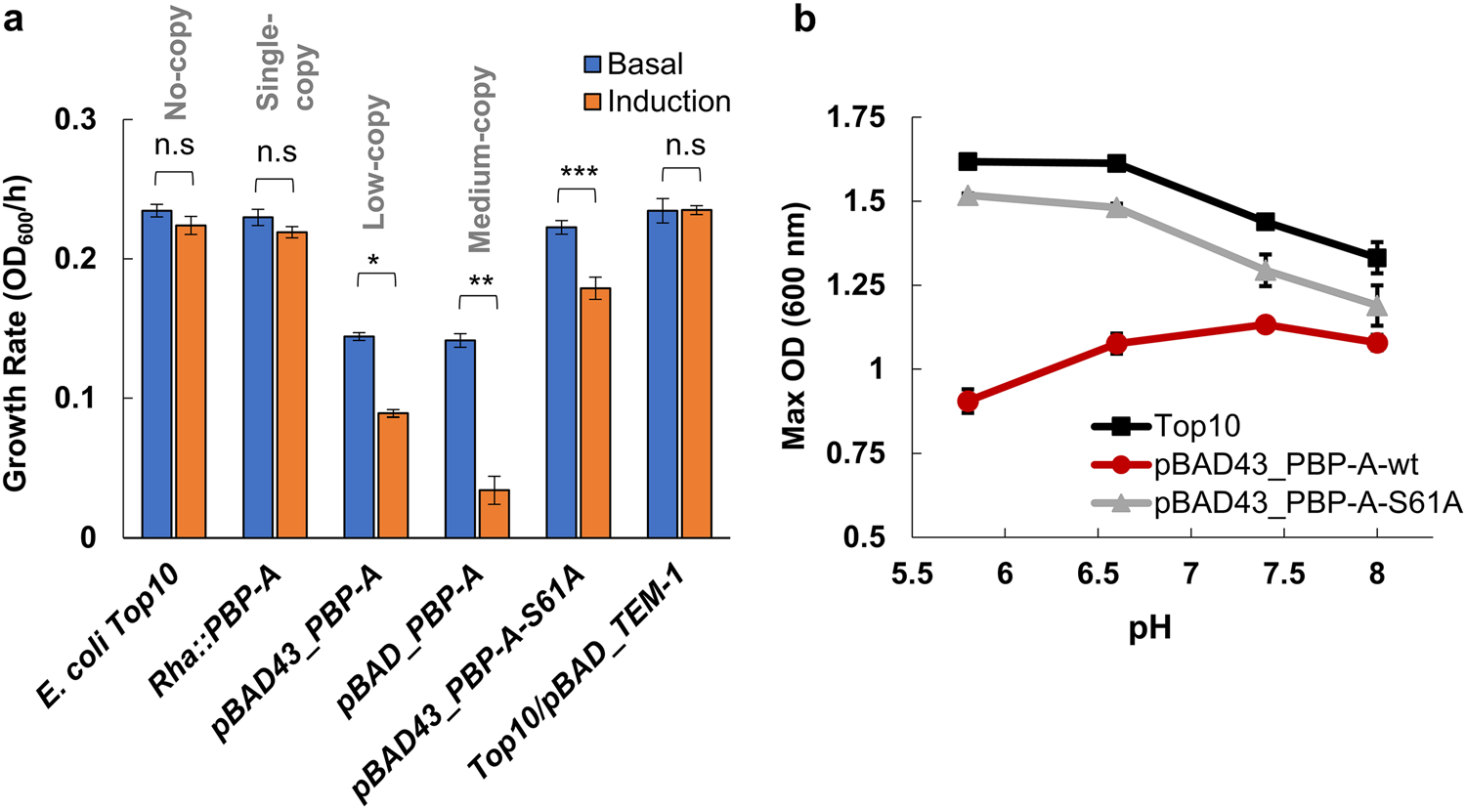
Fitness cost associated with the expression of PBP-A in the periplasm of *E. coli*. (**a**) Growth rates of different *E. coli* strains in unbuffered LB medium as estimated by the maximal slope of the optical density (OD) versus time curves. 0.5% (w/v) l-arabinose is added for induction under low- and medium-copy expression plasmids, while 0.5% (w/v) l-rhamnose is used with the chromosomal expression system. Average of three independent replicates with standard deviation (SD) as error bars. Asterisks indicate significant differences (*P < 1.4e-05, **P < 7.8e-05, ***P < 0.0013, *n* = 3, *t* test); n.s: not significant. **b)** Comparison of pH-growth profiles between *E. coli* TOP10 wild-type cells and cells expressing PBP-A-wt and PBP-A-S61A. Maximum optical density (max OD_600_) of *E. coli* cultures at different pH were measured to compare the effect of different pH. The maximum fitness cost due to PBP-A expression is detectable at acidic pH and the shift in optimum growth pH towards basic pH upon expression of PBP-A-wt is significantly detectable. For PBP-A-S61A the pH profile is similar to the wild-type *E. coli* cells. The data represent average of three replicates with standard deviations as error bars.

PBP-A was expressed in the periplasm of *E. coli* using various systems allowing to tune the expression level. To avoid mis-localization of PBP-A, we used a co-translational export to periplasm via the signal recognition particle (SRP) pathway by fusing *dsbA* signal sequence^23^ at the N-terminus of PBP-A. The gene was cloned in two arabinose-inducible plasmids, pBAD (pBR322 ori) and pBAD43 (pSC101 ori) featuring medium- and low-copy numbers, respectively. A single copy expression system was also built by inserting the gene in the polycistronic rhamnose operon of the *E. coli* genome. Here the gene is inserted in place of the second gene (*rhaA*) of the rhamnose operon and expression is induced by adding l-rhamnose. To estimate harmful effects, growth rates were compared by measuring the maximum slopes of growth curves after induction or under basal expression. As shown in Fig. 4a, growth defects are correlated with gene copy number and get worse upon promoter induction. Western blot analysis further confirmed the correlation between expression level, copy number and promoter induction (Supplementary Fig. S4). On the other hand, the expression of TEM-1 β-lactamase from the medium-copy pBAD plasmid was not associated with any detectable growth problem (Fig. 4a) even though both PBP-A and TEM-1 enzymes were expressed at similar levels as evaluated by Western blot with anti-histag antibody (Supplementary Fig. S5). This indicates that the reduced growth observed with PBP-A expression is not just the result of a general metabolic burden that would be associated with the production of a recombinant protein in the periplasm.

To investigate the possible implication of PBP-A activity, the catalytic mutant PBP-A-S61A was expressed from the low-copy pBAD43 vector. With this variant, a significantly lower growth defect was observed indicating that the catalytic activity was indeed associated with the deleterious effect. However, expression of the inactive enzyme still generates a small growth defect that may originate from protein misfolding or binding to the PG. Comparative Western blot analysis of the soluble and insoluble fractions after bacterial lysis indicates that, if any, only a minor fraction of PBP-A may precipitate in the periplasm (Supplementary Fig. S5). This analysis also confirmed the expected localization of the protein since we did not detect any signal corresponding to the pre-mature protein that may accumulate in the cytoplasm or in the membrane.

Since we discovered that DD-EPase activity of PBP-A on *E. coli’s* PG is increased at acidic pH, we hypothesize that it would correlate with an increased harm. To test that, we evaluated the pH effect by growing the cells in phosphate-buffered LB media at various pH ranging from 5.8 to 8. The cells were grown in LB until OD ~ 0.2, induced by arabinose, divided into four, and immediately buffered by adding concentrated buffers. As shown in Fig. 4b, acidic pH afforded a better growth for *E. coli* TOP10 while, for cells expressing PBP-As, the growth was strongly impaired under acidic growth, shifting the optimal pH to 7.5. This shift was not observed when expressing the catalytic mutant, confirming that PBP-A activity was responsible of this pH effect.

### In the periplasm of *E. coli*, PBP-A deteriorates the peptidoglycan and envelope integrity

**Figure 5:**
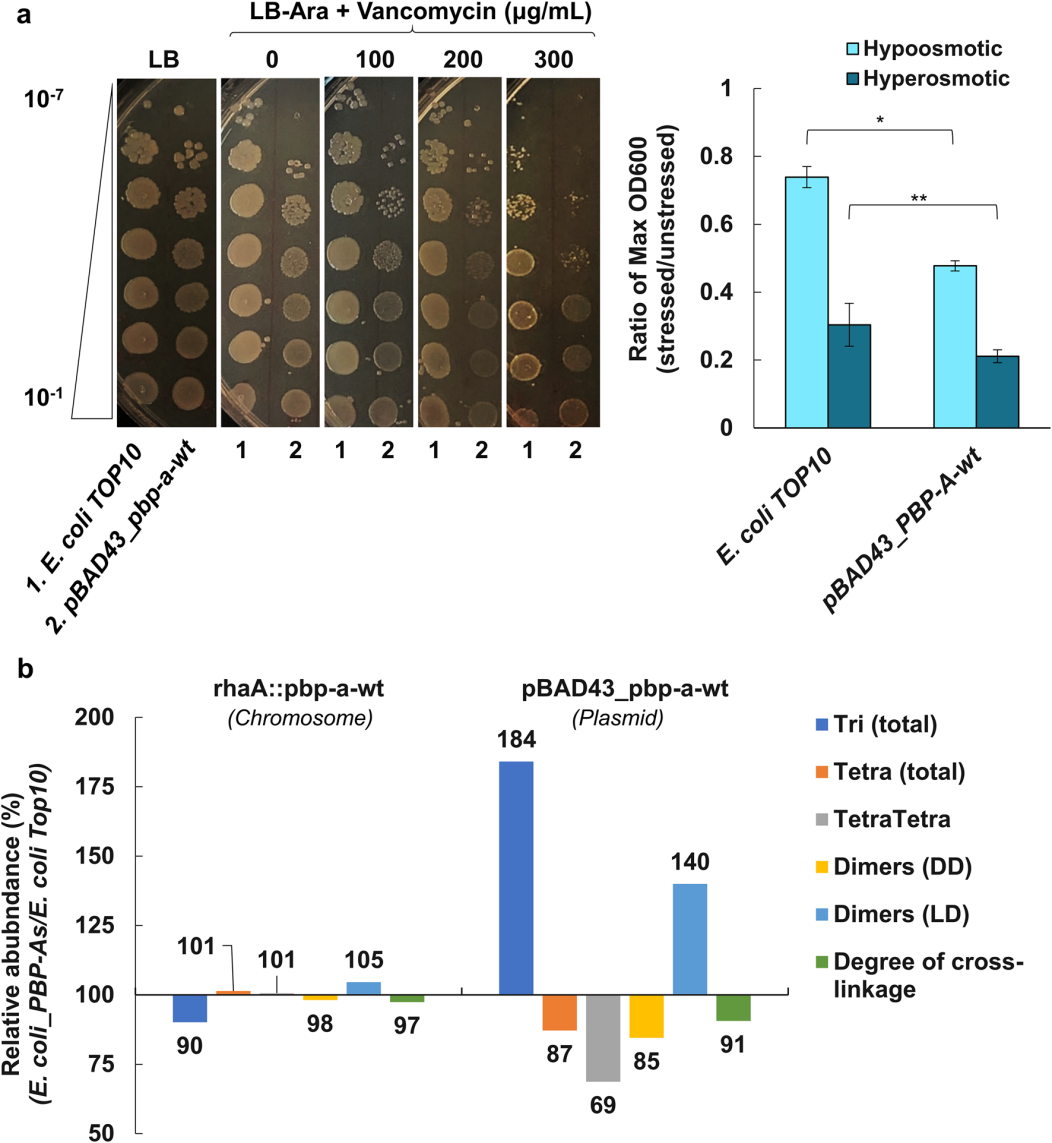
Envelope and peptidoglycan alterations upon expression of PBP-A in the periplasm of *E. coli* (**a**) Vancomycin susceptibility (left) and osmosensitivity (right) of *E. coli.* The bar chart represents ratio of maximum OD_600_ of cells expressing PBP-A under osmotic stress over unstressed cells (data obtained from three independent replicates, standard deviation as error bars). All the cultures are under induction of 0.5% (w/v) L-arabinose. LB media containing 0.75 M NaCl was used to apply the hyperosmotic stress and dilution (1:1) of the LB media by sterile distilled water was used to provide a hypoosmotic stress. The difference between *E. coli* TOP10 and PBP-A expressing cells were statistically significant for hypoosmotic stress (*P* < 0.005, n = 3, one-way ANOVA), while less significant for hyperosmotic stress (*P* < 0.09, n = 3, one-way ANOVA). (**b**) Comparative peptidoglycan analyses. Bars represent percentage of muropeptides detected in *E. coli* cells expressing PBP-A from single- and low-copy expression systems (*rhaA* locus in genome and pBAD43 plasmid respectively), relative to untransformed *E. coli* cells. While under single-copy, there is minimum change in PG composition, at low-copy expression from plasmid the PG is significantly altered: we observed a decrease in Tetra monomers, DD dimers and an overall decrease in PG cross-linkage, while Tri monomers and LD dimers are increasing. *E. coli* cells were induced for PBP-As expression at early exponential phase by addition of 0.5% (w/v) l-rhamnose or 0.5% (w/v) l-arabinose, for single- or low-copy expressions, respectively, and harvested at late exponential phase for PG isolation. The abundance (%) of all peptides detected in PG isolates of all the strains in this experiment are available in Supplementary Table S4.

We then evaluated the effect of PBP-A expression from the low-copy plasmid on two phenotypes that are associated with a damaged envelope, i.e. osmosensitivity^24^ and susceptibility to vancomycin, a glycopeptide antibiotic that is impermeant to the outer membrane of *E. coli* unless it is destabilized^1,25^. As shown in Fig. 5a, the presence of PBP-A in the periplasm indeed induces increased sensitivity to hypo- and hyper-osmotic pressures and to vancomycin.

Finally, we analyzed the chemical composition of the PG prepared from *E. coli* cells under single- and low-copy expression of PBP-A. Untransformed *E. coli* TOP10 strain was used as reference for PG profile and negative control. In Fig. 5b, the relative abundance of muropeptides that vary the most upon PBP-A expression are shown. The complete analysis is presented in Supplementary Table S4.

Expressing PBP-A from the low copy plasmid results in important PG alterations. More specifically, a reduction of PG crosslinking is observed, which is mainly detectable by the decrease in crosslinks between tetrapeptides (4–3 bridges or dd dimers). This further supports the dd-EPase activity of PBP-A that was detected in vitro with purified PG extracts (Fig. 3). Reduced crosslinking may also result from dd-CPase activity that would reduce the amount of pentapeptides that is the donor substrate of endogenous dd-TPases. However, pentapeptide abundance was very low in normal PG, preventing its quantification. Interestingly, 3–3 (or LD) crosslinks between two mDAP residues increased as well as total tripeptides, indicating an adaptation of the PG. Some other modifications were also detected including increase in tetra with glycine at position 4, which is another sign of PG adaptation and remodeling. Strikingly, when PBP-A was produced at very low level with the chromosomal expression system, the PG was barely altered suggesting that a damage threshold may be necessary for triggering PG remodeling.

## Discussion

*Thermosynechococcus elongatus* PBP-A belongs to a small family of low-molecular weight PBPs that are exclusively found and conserved in cyanobacteria^16^. In this study, we simulated the potential outcomes of *E. coli* acquiring PBP-A, observing some negative effects that led us to further explore its biochemical characteristics. Through this investigation, we found that the enzyme exhibits high specificity for amidated muropeptides with a d-iGln residue at position 2. To our knowledge, this represents first example of a dd-peptidase capable of discriminating a feature relatively remote from the catalytic site, with the nitrogen of the d-iGln amide being separated by 11 chemical bonds from the scissile peptide bond (Fig. 2a). Interestingly, amidation of carboxylic groups in the muropeptides of cyanobacterial peptidoglycan has been reported, although the identity of the amidated residues, whether d-iGlu and/or mDAP, was not determined^20^. Our results further suggest that the peptidoglycan of *T. elongatus*, at least, features amidation on the d-iGlu residue.

Interestingly, we also observed that PBP-A exhibits slight activity towards non-amidated substrates under acidic conditions. For the small, non-amidated peptide, the pKa of the alpha carboxylic group of d-iGlu is estimated to be around 4, akin to predictions for similar organic acids such as *N*-Acetyl-Alanine. Therefore, at pH 5, approximately 10% of d-iGlu molecules are expected to be in their protonated form. This likely accounts for the observed low-pH promiscuous activity of PBP-A, as a carboxylic acid, is more similar to an amide than a carboxylate in terms of charge distribution and hydrogen bonding pattern.

**Figure 6:**
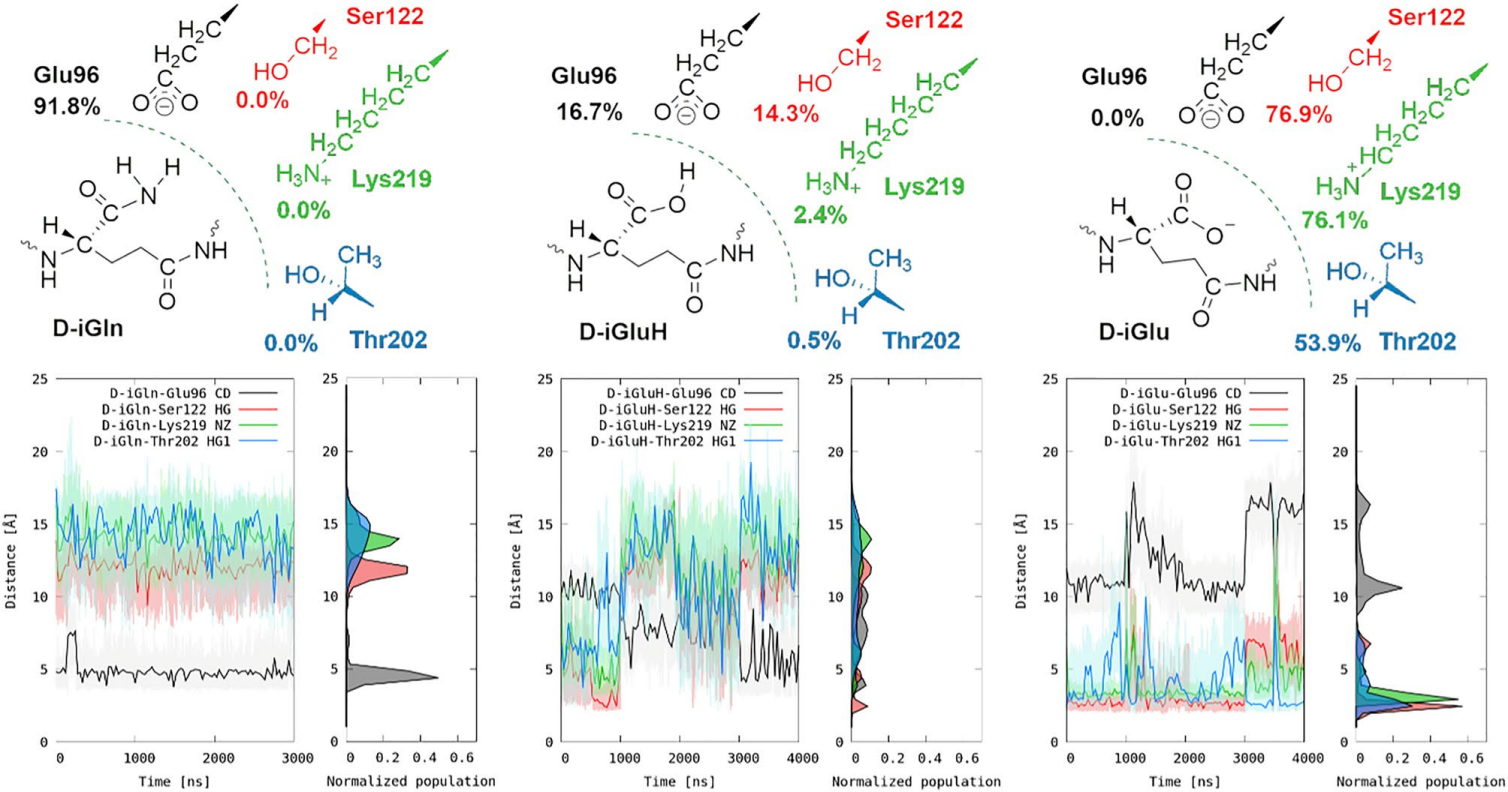
MD simulations predict different interactions between amidated (d-iGln), carboxylic (d-iGluH), and carboxylated (d-iGlu) substrate peptides and PBP-A active site. The upper row shows the hydrogen bonds interactions between the d-iGln, d-iGluH and d-iGlu and the key protein residues. % denotes contact percentage throughout the simulations. The bottom row shows the distances between the same set of residues and the d-iGln, d-iGluH and d-iGlu. Each 1000 ns in x axis of the time versus distance plots represent individual MD simulations/replicas that were merged together consecutively.

To investigate this hypothesis from a structural perspective, microsecond-long MD simulations were performed starting with a three-dimensional model of PBP-A (PDB ID: 2J8Y_X-RAY_)^17^ covalently acylated with a d-iGln-mDAP-d-Ala tripeptide on the catalytic serine residue through an ester bond with the C-terminal d-Ala (see details in Supplementary materials). In order to elucidate the interaction patterns and the key residues H-bond analysis was performed and were confirmed by distance analysis (Fig. 6). Interestingly, an almost permanent and long-lived interaction (91.8%) was observed between Glu96, at the active site entry, and the d-iGln (Fig. 6). Two hydrogen bonds involving the main chain NH and carboxamide NH_2_ groups of d-iGln as donors, and one oxygen of the Glu96 carboxylate as the acceptor, are predicted (see Supplementary Fig. S6). Furthermore, upon substituting d-iGln with d-iGlu, either in its protonated (carboxylic) or deprotonated (carboxylate) form, this interaction was observed to decrease or was not predicted, respectively (Fig. 6). Remarkably, negatively charged carboxylate model considerably alters the interaction pattern between d-iGlu and PBP-A (Ser122 (76.9%), Lys 219 (76.1%), and Thr202 (53.9%)). These interactions are absent in d-iGln model. It is important to note that the model featuring protonated d-iGlu aims to reflect the conditions at a mildly acidic pH (around 5–6), where carboxylic groups of both d-iGlu and Glu96 are partially protonated. Within this pH range, the concurrent protonation of d-iGlu and Glu96 should be minimal, and when only one proton is present, it may be equally positioned on the substrate or the enzyme. Overall, these predictions suggest that Glu96 is responsible for the observed discrimination between amidated and non-amidated muropeptides, aligning perfectly with both experimental observations of enzyme preference for d-iGln-containing peptide and low-pH promiscuous activity detected with d-iGlu-containing peptide (Fig. 2).

Assays performed on non-amidated PG extracts of *E. coli* confirmed dd-CPase activity observed with small peptides and further indicate presence of DD-endopeptidase (dd-EPase) activity resulting in the cleavage of isopeptidic bridges between the d-alanyl residue in position 4 and the d-stereospecific side chain of mDAP residue in position 3 of the crosslinked peptide. Promiscuous activities of LMW-PBPs were previously reported: among eight endopeptidase paralogues in *E. coli*, PBP4, PBP7 and AmpH serine hydrolase enzymes are endowed with both dd-CPase and dd-EPase activities, involved in maturation, remodeling and recycling of PG^26,27^. dd-TPase activity was also reported for PBP4 of *Staphylococcus aureus*^28,29^. Although promiscuity in PBPs is well established, we discovered an unprecedented pH-dependent substrate promiscuity on a relatively remote site from the peptidase active center. This is stressing out the potential importance of quite subtle peptidoglycan modifications for enzyme function and the high level of precision that underlies the peptidoglycan synthesis and maintenance.

Surprisingly, while the low-pH activation is observed for the dd-EPase activity on purified PG, it is not the case for the dd-CPase activity. There may be unidentified molecular determinants that are absent in the small peptide but affect differentially the pH-dependency of the promiscuous activities on the more complex PG substrate, but we cannot rule out that the observed dd-CPase activity on PG originates from a contaminating enzyme and not PBP-A. Indeed, if the real substrate of PBP-A is the amidated PG of cyanobacteria, we observe very low activity of PBP-A on *E. coli*’s PG, i.e. with high concentration of PBP-A where contamination of endogenous enzymes may interfere. This would also mean that the promiscuous dd-EPase of PBP-A on non-amidated PG is much higher than its dd-CPase activity. It would be potentially interesting to compare dd-CPase and DD-EPase activities of PBP-A on small amidated peptides to test if one activity is predominant over the other. Our strategy with mSDAP analog affording the stereochemical control of residue in position 3 could be adapted for synthesizing such bridged peptides.

In vivo, the low-pH dd-EPase activation of PBP-A on the non-amidated PG of *E. coli* correlates with a reduced PG cross-linkage and with pH-dependent harmful effects since the pH of the periplasm is not controlled by the bacteria and depends on the medium composition^30,31^. It should be noted that PBP-A is a positively charged protein below pH 7 (pI = 7.56) while the non-amidated PG of *E. coli* is negatively charged thanks to the presence of carboxylate functions mainly on residues 2 and 3 of the peptides and their C-terminus. Hence, PBP-A activity on the PG may be favored by electrostatic attraction. Indeed, preliminary results indicate fitness recovery at acidic pH when PBP-A is replaced by a variant featuring an acidic isoelectric point as a result of two surface charge inversions remote from the active site (Supplementary Fig. S7). This will have to be further investigated but such differential electrostatic effects have been reported in Gram positive bacteria expressing paralogous PG-hydrolases with acidic and basic pIs^32,33^. Interestingly, we noticed that homologous class A β-lactamases are often endowed with acidic isoelectric points. Since their biological role is to degrade β-lactam antibiotics, they must have evolved not to deteriorate the PG, and their acidic pI may be considered as a security feature.

In conclusion, we have demonstrated that PBP-A of *T. elongatus* is a dd-peptidase with a strong preference for peptides that are amidated in position 2. Promiscuous carboxypeptidase and endopeptidase activities on non-amidated substrates are exacerbated at acidic pH and responsible for activity-related and pH-dependent harmful effects associated with PG damage when the enzyme is expressed in the periplasm of *E. coli*. This may explain our unsuccessful endeavors to transform PBP-A into a β-lactamase and implies that directed evolution should be performed under protective environmental conditions that mitigate adverse effects (e.g. neutral pH, presence of osmoprotectants) and starting with an enzyme variant as harmless as possible to its host (e.g. high isoelectric point). From a broader perspective, our study highlights potential evolutionary constraints associated with enzyme promiscuity, in contrast with the accepted view of promiscuous activities as a springboard for enzyme neofunctionalization. Indeed, while promiscuous or generalist enzymes may be considered as attractive starting points for enzyme evolution, the organism’s capacity to tolerate such enzymes without excessive deleterious side-effects may be limited.

## Material and methods

### Strains and plasmids

*Escherichia coli* strain TOP10 was purchased from Invitrogen. pBad43 plasmid was kindly provided by Michael Deghelt from Institut de Duve, UCLouvain, Brussels.

#### Cloning of pbp-a gene in the rhamnose operon of the chromosome

The detailed protocol of genome engineering for building scarless gene libraries in RhaA locus of *E. coli* chromosome using CRISPR-Cas9 technology and λ-Red recombineering system is described in^34^. Briefly, recombinant *pbp-a* gene fused to DNA encoding the signal peptide of DsbA and a 6 × His tag fused at the amino- and carboxy-terminus, respectively, was replaced with the *rhaA* gene at the second position of the polycistronic operon of rhamnose in the genome of *E. coli*.

#### Cloning of pbp-a genes into low- and high-copy plasmids

The gene encoding *pbp-a* was amplified and assembled into pBAD43 plasmid thanks to the overlapping sequences integrated in primers followed by Gibson assembly. Following primer pairs were used for PCR amplification of *pbp-a* gene variants and pBAD43 and pBAD plasmid backbone. Overlapping sequences, upstream and downstream of *pbp-a* gene, are indicated as underlined and bold, respectively:

*Pair 1: pbp-a* fw: ttttagcgtttagcgcatcggcggc.

*pbp-a rv:* **ttggctttcgcccattcaactcagtg**atgatgatgatgg.

*Pair 2: pBAD-BackBone-PBPs fw*: **cactgagttgaatgggcgaaagccaa**tctagagtcgacctgcaggc.

*pBAD-BackBone-PBPs rv:* cgatgcgctaaacgctaaaactaaaccagccagcgccag.

The purified PCR products were inserted into pBAD plasmid backbones by mixing 15 μL of Gibson Master Mix, together with 50–100 ng of the linear vector backbone with a 2–3 folds molar excess of the insert, 0.5 μL of DpnI (to digest the remaining original (methylated) plasmid template), and ddH_2_O up to 20 μL. The assembly mix is then incubated for 1 h at 50 °C and the assembled fragments (circularized plasmid) can be detected on agarose gel. 1 μL of the assembly mix is electroporated into 50 μL of *E. coli* TOP10 electrocompetent cells (Invitrogen). Transformants are incubated at 37 °C, for 1 h before plating all the cells on LB plates containing spectinomycin and glucose (for repression of PBP-A expression) followed by overnight incubation 37 °C. Insertion of *pbp-a* genes in pBAD43 plasmid was verified by colony PCR and confirmed by sequencing of the PCR products from random clones. To avoid revertant selection and adaptation against toxicity of PBP-A, analyses were confirmed with fresh transformants and minimum freezing–thawing cycles.

Catalytic S61A mutant of PBP-A was prepared by site directed mutagenesis via QuickLib protocol^35^ using the following primers. The substitution and, overlapping sequences between primers are indicated as underlined and bold, respectively:

PBP-S61A_fw: AATGTCGGCG**GTGACCAAGTGTTTCCGG**CGGCCGCTACCATTAAATTCCCGATCCTGG.

PBP-S61A_rv: **CCGGAAACACTTGGTCAC.**

Lysine at positions 104 and 212 were mutated to Glutamic acid by incorporating mutations on primers followed by two PCR reactions on pBAD43_PBP-As template. Reaction 1 to amplify *pbp-A* gene from position 104 to 212 and reaction 2, for amplification of the remaining backbone of pBAD43_PBP-As. The PCR products of reaction 1 and 2 were purified and then assembled (thanks to overlap sequences incorporated into primers), by mixing 3–4 folds molar excess of the shorter fragment (reaction 1) with 50–100 ng of longer backbone (reaction 2) and 0.5 μL of DpnI (20 U/μL) into 15 μL Gibson master mix (100 mM Tris–HCl pH 7.5, 10 mM MgCl_2_, 0.2 mM of each four dNTPs, 1 mM NAD^+^, 15% (w/v) PEG-8000, T5 exonuclease (2 U/mL), Phusion DNA polymerase (33 U/mL), and Taq DNA ligase (1666 U/mL)), followed by transformation into *E. coli* competent cells.

Following primer pairs were used for the two reactions. Overlapping sequences, at regions 104 and 212 are indicated as underlined and bold, respectively:

Reaction 1

*Pbb-a_K104E fw:*

ATTGCCCCGGAAGCAGGCACCCTGCAGTATCAAGAACCGAATTCACAATACGCAG

*Pbb-a_K212E rv:* **CGGCAGCAGGGTATTGGTAACGGT**G.

Reaction 2

*Pbb-a_K212E fw:*

**ACCGTTACCAATACCCTGCTGCCG**GCCGGTCTGGGTGAAGGTGCAACGATCGCTCATAAAACC

*Pbb-a_K104E rv:* ATACTGCAGGGTGCCTGCTTC.

### Preparation of cell lysates

*Periplasmic extraction:* After induction of PBP-A expression for 3–4 h, *E. coli* cultures (10 mL) were harvested at 4400 × *g* for 10 min, supernatants were discarded, cells were resuspended in 0.33 mL of 20% (w/v) sucrose in 20 mM Tris–HCl, pH 8.0, and 33 µl of 0.1 M EDTA, pH 8.0 was added. Samples were incubated at room temperature for 10 min followed by 10 min centrifugation of cells at 6000×*g*. Supernatants were discarded, and the pellets subjected to osmotic shock by resuspending in 0.5 mL of 5 mM MgSO_4_. The mix was incubated at 4 °C for 10 min followed by 10 min centrifugation at 10,000×*g*. The supernatants were recovered as periplasmic extracts. The pellets could be used to recover the cytoplasmic content.

*Complete cell lysis:* Pellet of 10 ml bacterial culture was resuspended in 500 μL of lysis buffer containing 10% glycerol, 0.1% Triton X-100, 100 µg/mL lysozyme, 1 mM EDTA and 3 U DNAse. The cell suspension was incubated at room temperature for 30 min and sonicated 5 times for 20 s until the samples were no longer viscous and a clear lysate was obtained. After centrifugation at 4 °C and 12,000×*g* for 10 min, the supernatant was collected as the soluble cell lysate.

*Insoluble fraction:* The pellet remaining from the cell lysis preparation was considered as insoluble fraction and it was suspended in 500 μL of PBS, pH 7.0 for further analysis.

### Western blot analysis

Volumes of culture were normalized to the same OD600, by diluting the denser cultures, to subject similar numbers of cells to the periplasmic extraction. Protein samples are run on 4–20% iD PAGE Gel (Eurogentec) and transferred onto a 0.2 μm nitrocellulose membrane using the Trans-Blot^®^ Turbo™ Transfer System at constant 2.5 A and up to 25 V for 5 min. Then the membrane is incubated in blocking solution (5% non-fat dry milk, 0.2% Tween, PBS buffer pH 7.2) for 1 h at room temperature under gentle agitation to prevent non-specific antibody binding. The membrane is incubated for 1 h in primary antibody (5 mg/mL of rabbit anti-His (Sigma) diluted 1:2000 in blocking solution). The membrane is washed 3 times with the blocking solution for 10 min under agitation to remove unbound primary antibody. The tray is changed to decrease the background effect of primary antibodies and the membrane is incubated for 1 h in secondary antibody (Peroxidase AffiniPure Donkey Anti-rabbit IgG (H + L)), Jackson ImmunoResearch, UK). Before proceeding to detection, the membrane is washed 2 times with the blocking solution for 10 min under agitation followed by one time washing with PBS buffer. For chemiluminescence detection of signals, the membrane is soaked in substrates mixture of substrate A and substrate B (Pierce™ ECL Plus Western Blotting Substrate, Thermofisher) with a ratio of 40:1 as suggested by supplier. The signals are detected on Amersham™ Imager 600 (GE Healthcare).

### Purification of PBP-A proteins

PBP-A proteins were purified from total soluble lysate by affinity chromatography thanks to the 6-histidine (6 × His) tag fused to the C-terminal. Briefly, the total soluble lysate of *E. coli* cells expressing different PBP-As were loaded onto a Ni-Sepharose column. After several washes by washing buffer (20 mM HEPES, 20 mM Imidazole, 150 mM NaCl, pH 8.0) 4–5 times the column volume, a stepwise gradient of imidazole (50–500 mM in 50 mM steps) was applied to elute the variants.

### Synthesis of mSDAP-containing peptides

Pentapeptide substrates were synthesized by solid-phase peptide synthesis (SPPS) using standard fluorenylmethoxycarbonyl (Fmoc) chemistry on a Symphony synthesizer (Protein Technologies Inc): Ac-l-Ala-d-iGlu-l-Cys-d-Ala-d-Ala-OH and Ac-l-Ala-d-iGln-l-Cys-d-Ala-d-Ala-OH. Pre-loaded Fmoc-d-Ala-Wang resin (Novabiochem) was used as the support. To modify l-cystein into mSDAP aminoacid, peptides were mixed with a 25-fold excess of H-β-Chloro-d-Ala-OH (Bachem) in water (pH 8 with *N*-Methylmorpholine) and reacted for 4 h at 45 °C. The peptides obtained were purified by reverse-phase HPLC to > 98% purity and characterized by mass spectrometry.

### PBP-A activity assay using synthetic peptides as substrates

A reaction containing 5 μL of enzymes (0.15–0.2 mg/mL), 10 μL of 5 mM synthetic pentapeptides (Acetyl-l-Ala-d-iGlu-mSDAP-d-Ala-d-Ala or Acetyl-l-Ala-d-iGln-mSDAP-d-Ala-d-Ala) and 10 μL HEPES buffer (20 mM, pH 7.4), incubated for 1 h at room temperature, followed by analysis by nanoUPLC-MS. For inhibition of PBP-A, 10 μL of 100 mM penicillin G (PenG), prepared in 20 mM HEPES pH 7.4, was used instead of the buffer in the above-mentioned reaction.

### Analysis of peptides by nanoUPLC-MS

In-solution samples are prepared by adding 1% trifluoroacetic acid (TFA) to the samples to reach pH below 7.0.

#### Peptide separation using nanoUPLC

Peptide mixture was separated by reverse phase chromatography on a NanoACQUITY UPLC MClass system (Waters) working with MassLynx V4.1 (Waters) software. 5 μL of each were injected on a trap C18, 100 Å 5 μm, 180 μm × 20 mm column (Waters) and desalted using isocratic conditions with at a flow rate of 15 μL/min using a 99% formic acid and 1% (v/v) ACN buffer for 3 min. Peptide mixture was subjected to reverse phase chromatography on a C18, 100 Å 1.8 μm, 75 μm × 150 mm column (Waters) PepMap for 35 min at 35 °C at a flow rate of 300 nL/min using a two parts linear gradient from 1% (v/v) ACN, 0.1% formic acid to 40% (v/v)) ACN, 0.1% formic acid for 15 min and from 40% (v/v) ACN, 0.1% formic acid to 85% (v/v)) ACN, 0.1% formic acid for 10 min. The column was re-equilibrated at initial conditions after washing 30 min at 85% (v/v)) ACN, 0.1% formic acid at a flow rate of 300 nL/min. For online LC–MS analysis, the nanoUPLC was coupled to the mass spectrometer through a nano-electrospray ionization (nanoESI) source emitter.

#### LC-QTOF-MS/MS analysis (DDA)

DDA (Data Dependent Analysis) analysis were performed on an SYNAPT G2-Si high definition mass spectrometer (Waters) equipped with a NanoLockSpray dual electrospray ion source (Waters). Precut fused silica PicoTip^R^ Emitters for nanoelectrospray, outer diameters: 360 μm; inner diameter: 20 μm; 10 μm tip; 2.5″ length (Waters) were used for samples and Precut fused silica TicoTip^R^ Emitters for nanoelectrospray, outer diameters: 360 μm; inner diameter: 20 μm; 2.5″ length (Waters) were used for the lock mass.solution. The eluent was sprayed at a spray voltage of 2.8 kV with a sampling cone voltage of 25 V and a source offset of 30 V. The source temperature was set to 80 °C. The cone gas flow was 20 L/h with a nano flow gas pressure of 0.4 bar and the purge gas was turned off. The SYNAPT G2Si instrument was operated in DDA (data-dependent mode), automatically switching between MS and MS2. Full scan MS and MS2 spectra (*m*/*z* 50-2000) were acquired directly after injection to 35 min in resolution mode (20,000 resolution FWHM at *m*/*z* 400) with a scan time of 0.1 s. Tandem mass spectra of up to 10 precursors were generated in the trapping region of the ion mobility cell by using a collision energy ramp from 17/19 V (low mass, start/end) to up to 65/75 V (high mass,start/end). Charged ions (+ 1, + 2, + 3, + 4) are selected to be submitted to the MS/MS fragmentation over the m/z range from 50 to 2000 with a scan time of 0.25 s. For the post-acquisition lock mass correction of the data in the MS method, the doubly charged monoisotopic ion of [Glu^1^]-fibrinopeptide B was used at 100 fmol/μL using the reference sprayer of the nanoESI source with a frequency of 30 s at 0.5 μL/min into the mass spectrometer.

#### ESI-QTOF data processing

Data were processed with MassLynx V4.1(Waters). Spectrum for each sample were extracted from the TIC (Total Ion Chromatogram) and submitted to a search of masses of interest.

### Activity assay on purified PG or muropeptides of *E. coli*

HPLC based activity assays were carried out in a final volume of 50 µL and in buffer containing 20 mM buffer agent (HEPES or NaAcetate), 100 mM NaCl, 0.05% (w/v) Triton-X 100 (reduced), 10 µL of the substrate and 5 µM of protein(s). HEPES/NaOH was used for reactions at pH of 7.5, NaAcetate was used for reactions at pH 5.0. The reaction mixture was incubated for 2 h in a thermoshaker at 37 °C, 900 rpm for the indicated time. The reaction was stopped by boiling the samples for 10 min at 100 °C. For standard activity assays on PG, purified PG sacculi was added to the reaction mixture and after the incubation the cellosyl digestion followed overnight. Activity assays on muropeptides were performed on predigested PG sacculi with cellosyl. The pH value of the muropeptides was adjusted before they were added to the reaction mixture. The reaction products were reduced with sodium borohydride and analyzed by reversed phase HPLC (see below).

#### Assays of PBP-As with PG of *E. coli*

PG from the *E. coli* TOP10 and CS703-1 strains were isolated as previously published^36^. A mixture containing 20 mM NaAcetate pH 5.0, 100 mM NaCl, 0.05% (w/v) Triton X-100 and 5 µM of each PBP-A protein was incubated for 2 or 6 h at 37 °C. After boiling, the samples were digested overnight with cellosyl, reduced with sodium borohydride, and the muropeptides were analyzed by reversed phase HPLC (see below).

### Preparation of PG and muropeptides (cellosyl products)

PG sacculi were isolated from *E. coli* cells as described^36^. Purified PG from *E. coli* CS703-1 and BW25113∆6LDT were mixed with cellosyl (0.5 µg/mL) in 80 mM sodium phosphate, pH 4.8 and incubated overnight in a thermal shaker at 37 °C and 900 rpm. The reaction was stopped by boiling and the sample was centrifuged for 10 min at 10,000×*g* (ambient temperature) to separate the soluble muropeptides (supernatant) from insoluble material. The muropeptides were either used as substrate in enzymatic activity assays or analyzed by reversed phase HPLC.

### Reduction of muropeptides with sodium borohydride

Prior to HPLC analysis, muropeptides were reduced with sodium borohydride^36^. Muropeptides were mixed with the same volume of 0.5 M sodium borate pH 9.0 in a 2 mL Eppendorf tube with a punctured lid. The reduction process was started by adding ~ 1 mg of sodium borohydride powder with a spatula. The samples were centrifuged for 20 min at 2000×*g* at ambient temperature during the reduction. The pH value of the samples was adjusted to 4–5 with phosphoric acid, the sample was centrifuged (5 min, 12,000×*g*, ambient temperature) and the supernatant was analyzed by HPLC.

### Reversed-phase HPLC analysis of muropeptides

Reduced muropeptides were separated on a Prontosil 120-3-C18-AQ 3 µm reversed-phase HPLC column (Bischoff) as described^36^. The column was pre-equilibrated with solvent A (50 mM sodium phosphate, pH 4.31, 0.0001% NaN_3_). Muropeptides were separated at 55 °C with a 90 min linear gradient to solvent B (75 mM sodium phosphate, pH 4.95, 15% (or 30% methanol to facilitate elution of all anhydromuropeptides)). Muropeptides were detected by UV absorbance at 205 nm and analyzed by the Laura V4.2.11.129 software (LabLogic System Ltd.). Fractions were collected during the HPLC run and analyzed by tandem mass spectrometry (MS/MS) as described previously^37^.

### Bacterial growth rate measurements

For all the bacterial cultures in this study, pre-cultures were started by inoculation of cells from a single colony into 5 ml of media containing appropriate antibiotics and 0.4% (w/v) d-glucose (for repression of leaky recombinant protein expression) and incubated overnight at 37 °C under agitation of 180 rpm.

To start the growth measurement experiment, 1 ml of pre-culture was inoculated into 100 ml of LB media without any antibiotic in a sterile 500 ml erlenmeyer flask and incubated at 37 °C under agitation of 180 rpm. When cultures reached early exponential phase (OD600 ~ 0.2), cultures were divided into half by transferring each 50 ml to a sterile 250 ml erlenmeyer. One of the cultures of each strain was induced for expression of PBP-As by adding l-arabinose (0.5%, w/v) followed by incubation for 4–5 h. OD_600_ of the cultures were measured over time from the start of the cultures.

To estimate fitness effect associated with expression of PBP-As in *E. coli* TOP10, we measured growth rate after induction of PBP-As expression and maximum optical density at 600 nm (max OD_600_) from growth curves of induced and basal expressions. Growth rate of each culture was estimated by measuring the slope of the growth curve in exponential phase and max OD of the cultures were simply determined as highest value(s) of absorbance at 600 nm during the growth. Fitness cost of each strain are quantified by dividing average values of growth rate and OD_max_ of induced cultures to the basal (non-induced) cultures as shown in following formula:

$$Ratio\, of_{{{\text{ growth rate}},{\text{ OD max}}}} = \frac{{ induced\, cultures_{{{\text{growth rate}},{\text{ OD max}}}} }}{{basal\, cultures_{{{\text{growth rate}},{\text{ OD max}}}} }}$$

The growth rate, can be estimated by the slope of the tangent line drawn to the inflexion of the growth curve at exponential phase, which can be estimated by following equation in Microsoft excel:

$$b = \frac{{\sum \left( {x - \overline{x}} \right)\left( {y - \overline{y}} \right)}}{{\sum \left( {x - \overline{x}} \right)^{2} }}$$

where *b* is the slope of the regression line and *y* values are absorbance at 600 nm (OD_600_) measured at time points *x*.

### Osmotic stress assays

*Hyperosmotic stress:* LB growth medium was replaced with LB supplemented with high concentration of NaCl (0.75 M) and the growth was monitored over time. Growth rate and max OD_600_ were estimated as explained in previous section.

*Hypoosmotic shock:* LB growth medium was diluted by addition of sterile distilled water to growth media (1:1 dilution) at mid-exponential growth (OD_600_ ~ 0.4–0.5) followed by monitoring growth over time and calculation of growth rates and max OD_600_.

### Vancomycin susceptibility spotting assay

Cultures of different bacterial strains were induced for expression of PBP-As by adding 0.5% (w/v) l-arabinose at OD_600_ ~ 0.2–0.3 and grown to late exponential phase (OD_600_ ~ 0.6–0.7). The culture volumes were normalized to the same OD_600_ by diluting higher density cultures with LB media. From the OD_600_ normalized samples, cultures were diluted by series of sequential tenfold dilutions in sterile LB media up to 10^−7^ and then 4 μL of each dilution were spotted on LB agar supplemented with 0, 100, 200 and 300 mg/L of vancomycin and 0.4% (w/v) l-arabinose. Plates were incubated 16–20 h at 37 C.

### Growth of *E. coli* cultures under different pH

From pre-cultures, 250 mL cultures were started in LB media, grown until OD ~ 0.2, induced with 0.5% (w/v) l-arabinose, divided into 50 mL in different Erlenmeyer flasks, and immediately buffered by adding mixture of KH_2_PO_4_ and K_2_HPO_4_ of concentrated 1 M solutions in order to achieve LB medium with 50 mM total phosphate buffer at different pH (pH 5.8 to pH 8.0). The cultures were incubated at 37 °C under 180 rpm gentle agitation in and OD_600_ was measured every 20 min. The maximum OD_600_ was measured from as the highest value of absorbance reached during the growth in 18 h of growth.

### Isolation of *E. coli* cells lysate for peptidoglycan analysis

From overnight pre-cultures, 4 mL was inoculated into 400 mL of LB media without any antibiotic in a sterile 2 L Erlenmeyer flask and incubated at 37 °C under agitation of 180 rpm. At early exponential phase (OD_600_ ~ 0.2), cultures were induced for expression of PBP-As by adding 0.5% (w/v) l-arabinose followed by incubation to the late exponential state (OD_600_ ~ 0.7). The cultures were rapidly cooled in an ice/water bath to 4C and cells were harvested by centrifugation at 4C, 5000 rpm. The supernatants were discarded, and each pellet was resuspended in 6 mL of ice-cold water. Each cell suspension was slowly (dropwise) added into 6 mL of pre-boiling 8% SDS solution under gentle stirring on a magnetic stirrer in an Erlenmeyer flask. Note that drop-wise addition of cell suspension into boiling SDS is important to ensure rapid disintegration of the cells that is required for the fast inactivation of the endogenous autolysins. Samples were boiled under stirring for 30 min. A few drops of water were added if excess foam developed or if the volume of the boiling solution decreased too much. The suspension should boil at low heat all the time. Finally, samples were cooled down to room temperature and transferred to Falcon tubes and kept at room temperature until further treatment and HPLC–MS analysis.

### Docking and simulation details

For molecular dynamics (MD), we chose to simulate the structure of PBP-A covalently acylated with d-iGln-mDAP-d-Ala tripeptide as a minimal substrate for exploring the binding mode of d-iGln in the active site. We did not extend further the substrate (which is naturally polymeric) for minimizing calculation time and chose to run simulations with the acyl-enzyme intermediate because it is introducing a covalent constraint between the enzyme and the substrate, imposing the proximity between the last d-Ala residue and the catalytic Ser61. This constraint is minimizing conformational freedom of the peptide, therefore maximizing prediction confidence. Moreover, following the Hammond postulate, an intermediate is generally considered as more similar to the transition state than the enzyme substrate complex, so it is more relevant from a catalytic perspective.

Protein, ligand, and complex (without bond between Oγ of Ser61 and carbon atom of d-Ala of the ligands) was used in the docking procedure. The structure of PBP-A in complex with a penicillin G compound (PDB ID: 2J8Y)^17^ was used as the protein model. Tripeptide models were built as described in the following paragraph. Docking was performed for each system and 10.000 poses were obtained by using Rosetta 3.13^38^ and the following protocol^39^ using a Monte Carlo (MC) based multi-scale docking algorithm was employed. Backbone flexibility of protein active site was provided using backrub algorithm and hard_rep score.

500 poses with the lowest interface energy were initially selected and near attack conformation analysis (NAC) was performed with the distance criteria: ≤ 3.7 Å and angle: 100° ≥ and ≤ 130°. The poses which met the criteria were listed (nucleophilic attack criteria was also considered). As a result, the following number of poses were obtained: amidated tripeptide—117 poses, carboxylated tripeptide—6 poses, and carboxylic tripeptide—39 poses. Two models with lowest interface energy and two models with the lowest distance between Oγ atom of Ser61 and the carbon atom of d-Ala for each system were selected for further studies (Supplementary Table S7).

Initial reference structure was retrieved from the Protein Data Bank (PDB ID: 2J8Y_X-RAY_)^17^ and unnecessary ligands were removed from the initial structures. The reference structure used in this study were protonated to pH of 7.4 using H++ server^40^. The protonation states of crucial residues were evaluated across a wider pH range (4.0–7.4) to determine their pH range for protonation and deprotonation. Tripeptide models (amidated, carboxylated, and carboxylic) were built from the reference structure using tLeaP package from Amber22 program package^41^. 4 fs time step was used for production runs using hydrogen mass repartitioning (HMR) implemented in ParmED (Supplementary Table S8)^41,42^. The Roe protocol was used which includes five-step minimization and a four-step equilibration protocol^43^. 1 µs long MD simulation with 300 K and NPT ensemble utilizing Monte Carlo barostat^44^ and Langevin temperature coupling^45^ with a gamma of 1.0 ps^−1^ was performed for each system. All MD simulations were performed utilizing the Amber22 program package^41^. AMBER ff19SB force field^46^ and TIP3P^47^ water model were used, and the protein structure was immersed in a truncated octahedral box with a distance at least 16 Å^41^.

In force field parameterization, the terminal sides of d-iGln, d-iGluH, d-iGlu, mDAP and SAP were capped with ACE and NME in order to provide charge stabilization and mimic the neighboring residues. In the substrate, acetylated d-iGln (capped with ACE) was used since there is no N-terminal there in the natural polymeric substrate. d-Ala and Ser61 were parameterized together and was named SAC. (Supplementary Table S9). The optimization calculations were performed at the HF/6-31G(d) level of theory using Gaussian 16 program package (Rev C.01)^48^. The restrained electrostatic potential (RESP) charges were obtained using the Merz–Singh–Kollman (MK) scheme (Iop: (6/33 = 2, 6/42 = 6, 6/50 = 1)) at the HF/6-31G(d) level of theory.

### MD analysis details

All analyses were performed with the cpptraj^49^ module of Amber22 program package^41^. The backbone root-mean-square deviation (RMSD) analysis was performed using the backbone atoms of all models with respect to initial structure before MD simulation. The distance criteria for H-bond analysis were defined as ≤ 3.2 Å based on heavy atom distances (acceptor to donor heavy atom), and the angle cutoff is 135°. The contact percentage (%) in H-bond analysis is defined as the percentage of total contacts of the residues throughout simulations. The analysis was performed among active site residues, namely Ser61-Lys64, Ser122-Asn124, Leu158, Lys219-Asp222, and all enzyme residues including tripeptides. Clustering analysis was performed using the HierAgglo algorithm (10 clusters, linkage, based on the Cα RMSD of enzyme residues in order to present representative structure in visualization. Sieve (50, random) was also utilized^50^. “C” denotes to the cluster. Graphs was plotted by Gnuplot (version 5.4.4)^51^, ChimeraX^52^ and PyMOL (version 2.5.4)^53^ were used for the visualization and illustration of the studied models.

### Supplementary Information

Supplementary Information.

## Data Availability

Data is provided within the manuscript or supplementary information files.
