# Supplementary material for: PBP-A, a cyanobacterial dd-peptidase with high specificity for amidated muropeptides, exhibits pH-dependent promiscuous activity harmful to Escherichia coli
Source: Sci Rep. 2024 Jun 18;14:13999. doi: 10.1038/s41598-024-64806-x (PMC11189452; doi:10.1038/s41598-024-64806-x)
Supplement: Supplementary file 1 — Supplementary Information. [file 41598_2024_64806_MOESM1_ESM.docx]

**Supplementary Materials**

**PBP-A, a cyanobacterial DD-peptidase with high specificity for amidated muropeptides, exhibits pH-dependent promiscuous activity harmful to *Escherichia coli***

Gol Mohammad Dorrazehi, Matthias Winkle, Martin Desmet, Vincent Stroobant, Gamze Tanriver, Hervé Degand, Damien Evrard, Benoît Desguin, Pierre Morsomme, Jacob Biboy, Joe Gray, Karolina Mitusińska, Artur Góra, Waldemar Vollmer and Patrice Soumillion

**List of Supporting Information**

**Details of computational study**

**Supplementary Tables**

**Supplementary Table S1.** Relative abundance of muropeptides detected by HPLC in assays of PBP-A with purified PG from *E. coli* BW25113∆6LDT.

**Supplementary Table S2.** Relative abundance of muropeptides detected in assay of PBP-A on PG extracts of *E. coli* CS703-1 at pH 7.5 and 5.0.

**Supplementary Table S3.** Relative abundance of muropeptides detected in assay of PBP-A on PG extracts of *E. coli* TOP10 at pH 7.5 and 5.0.

**Supplementary Table S4.** Relative abundance (%) of muropeptides detected in PG isolated from *E. coli* expressing PBP-A*.*

**Supplementary Table S5.** The RMSF values for the catalytic Ser61 and the tripeptide units for the PBP-A in complex with the substrate in amidated, carboxylic (COOH), and carboxylated (COO^-^) form.

**Supplementary** **Table S6.** Clustering summary for the systems bearing amidated, carboxylic and carboxylated tripeptides.

**Supplementary Table S7**. List of models used for MD simulations.

**Supplementary Table S8.** Summary of the initial setup.

**Supplementary Table S9.** Prepin files for the parameterized tripeptide units**/**residues.

**Supplementary Figures**

**Supplementary Figure S1**. Capillary electrophoresis analysis of PBP-A assay with amidated pentapeptide.

**Supplementary Figure S2**. HPLC peak detection in assay of PBP-As with purified PG

**Supplementary Figure S3.** Assaying PBP-A wt and catalytic mutant PBP-A-S61A on muropeptides from *E. coli*. For these experiments, the PBP-A enzymes were purified from a cytoplasmic expression system.

**Supplementary Figure S4**. Western blot analysis for evaluating the effect of copy number on expression level of PBP-A.

**Supplementary Figure S5**. Translocation of proteins into periplasm of *E. coli*. Evaluation of signal peptide cleavage and PBP-A in vivo solubility.

**Supplementary Figure S6.** Representation of key H-bond interactions between D-iGln and Glu96 (representative structure from cluster C0).

**Supplementary Figure S7.** Fitness cost is stronger under acidic conditions and can be mitigated by lowering the isoelectric point of PBP-A.

**Supplementary Figure S8**. Backbone RMSD for the protein (right panel) and tripeptide (middle panel) with respect to first structures after equilibration for PBP-A in complex with (a) amidated, (b) amidated conformer with the lowest distance, (c) carboxylic (COOH), and (d) carboxylated (COO^-^) tripeptides.

**Supplementary Figure S9.** Distance plot between (a) D-iGln@NE2 - Glu96@CD and (b) D-iGln@H - Glu96@CD followed by corresponding histograms (c) and (d).

**Supplementary Figure S10**. Representation of the tripeptide orientations for each model (representative structure from cluster C0 for each system).

**Details of computational study**

This study covers understanding conformational behaviors and investigation of the interaction patterns between the tripeptides, particularly D-iGln, and Glu96 of PBP-A (D-iGln-mDAP-DAla-O-enzyme). Microsecond-long MD simulations were performed using diﬀerent starting velocities. The stability of the studied systems and interactions between the Glu96 of PBP-A and D-iGln were explored. Moreover, H-bond and distance analyses, along with clustering were combined to elucidate interaction patterns and to give insight activity difference between tripeptide models. Initially, the stability of the studied systems were investigated using backbone RMSD (root mean square deviation) and RMSF (root mean square fluctuation) analysis. The backbone RMDS and RMSF displayed that all systems are stable during the simulations (**Supplementary Figure S8 and Supplementary Table S5**). H-bonds interaction contributes to stability of complex systems. In order to elucidate the interaction patterns between the amidated tripeptide with PBP-A, particularly with D-iGln, H-bond analysis was performed. In one of four MD simulations in amidated system belonging to the lowest distance models, an almost permanent and long-lived interaction between Glu96 and D-iGln (86.5%) was observed. In order to assess the persistency of this interaction, and provide statistically significant findings, we increased the sampling, and performed two more independent simulations for this model. The H-bonds analyses showed that the interaction between Glu96 and D-iGln are persistent (long-lived) (91.8%). Additionally, this interaction was tracked and confirmed throughout the simulations by using distance analysis (**Supplementary Figure S9**).

Furthermore, the differences in interaction patterns for the models with the amidated, carboxylic and carboxylated tripeptides were further investigated. In order to provide consistency between tripeptide atoms on D-iGln/D-iGluH/D-iGlu, side chain carbonyl carbon and the corresponding atoms in PBP-A residues were examined by H-bond and distance analysis (see main manuscript).

Lastly, cluster analysis was performed in order to obtain the most persistent conformation of the PBP-A in complex with the amidated tripeptide along with the other tripeptides. 10 clusters were investigated and mostly populated cluster zero (C0), accounting for more than 95.5% of the conformations was identified as a representative cluster and was used for visualization of interaction patterns between D-iGln and Glu96 and orientation of the tripeptides towards PBP-A (**Supplementary Figures S6-S10 and Supplementary Table S6**). While amidated and carboxylic tripeptides mostly towards omega loop side, carboxylated tripeptides more towards hinge 2 region (**Supplementary Figure S10**). This observation suggests that negatively charged peptides prefers different orientations, conceivably different interactions.

**Supplementary Tables S1.** Relative abundance of muropeptides detected by HPLC in assay of PBP-A with purified PG from *E. coli* BW25113∆6LDT*.*

**Supplementary Table S2.** Relative abundance of muropeptides detected in assay of PBP-A on PG extracts of *E. coli* CS703-1 at pH 7.5 and 5.0.


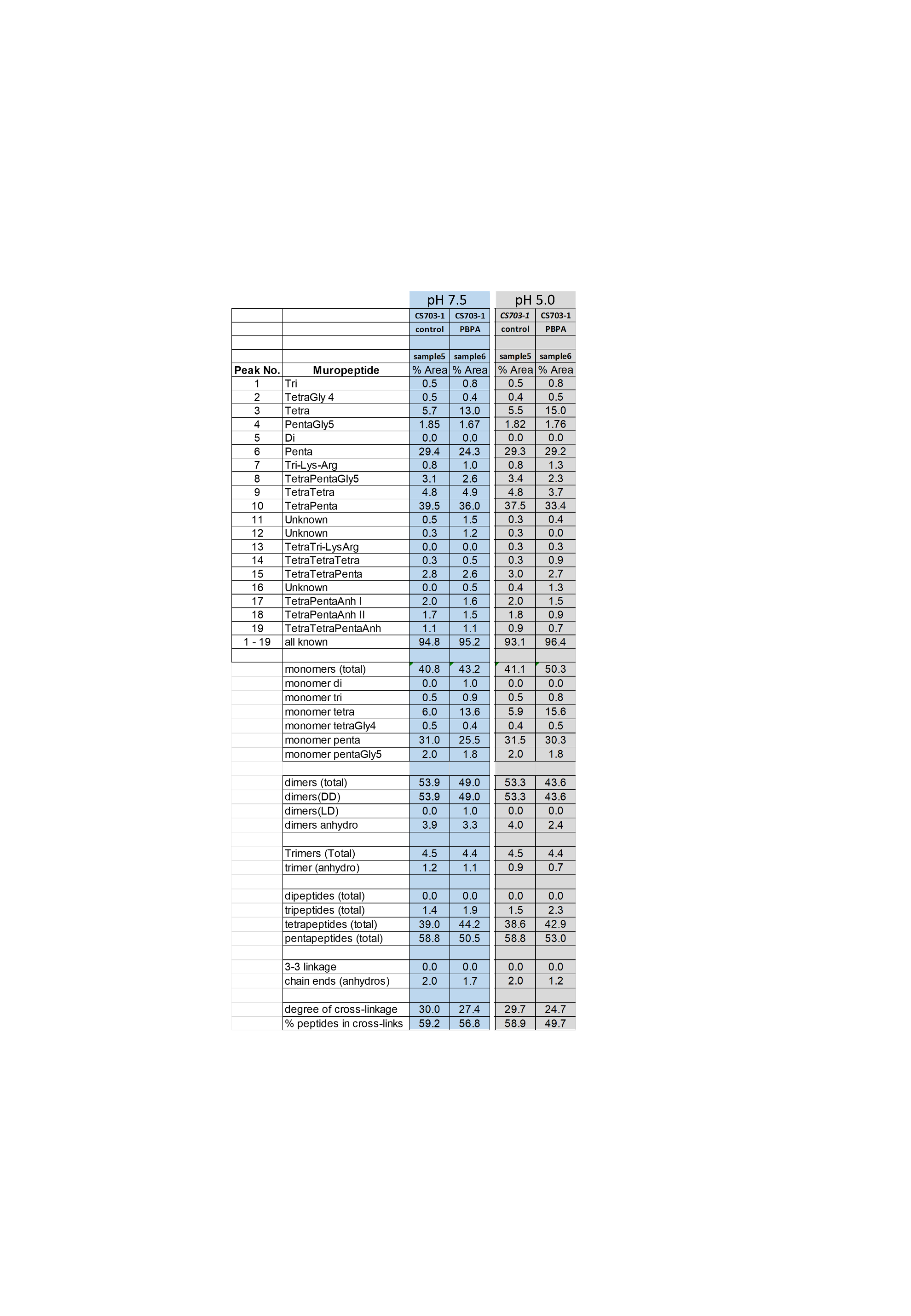


**Supplementary Table S3.** Relative abundance of muropeptides detected in assay of PBP-A on PG extracts of *E. coli* TOP10 at pH 7.5 and 5.0.


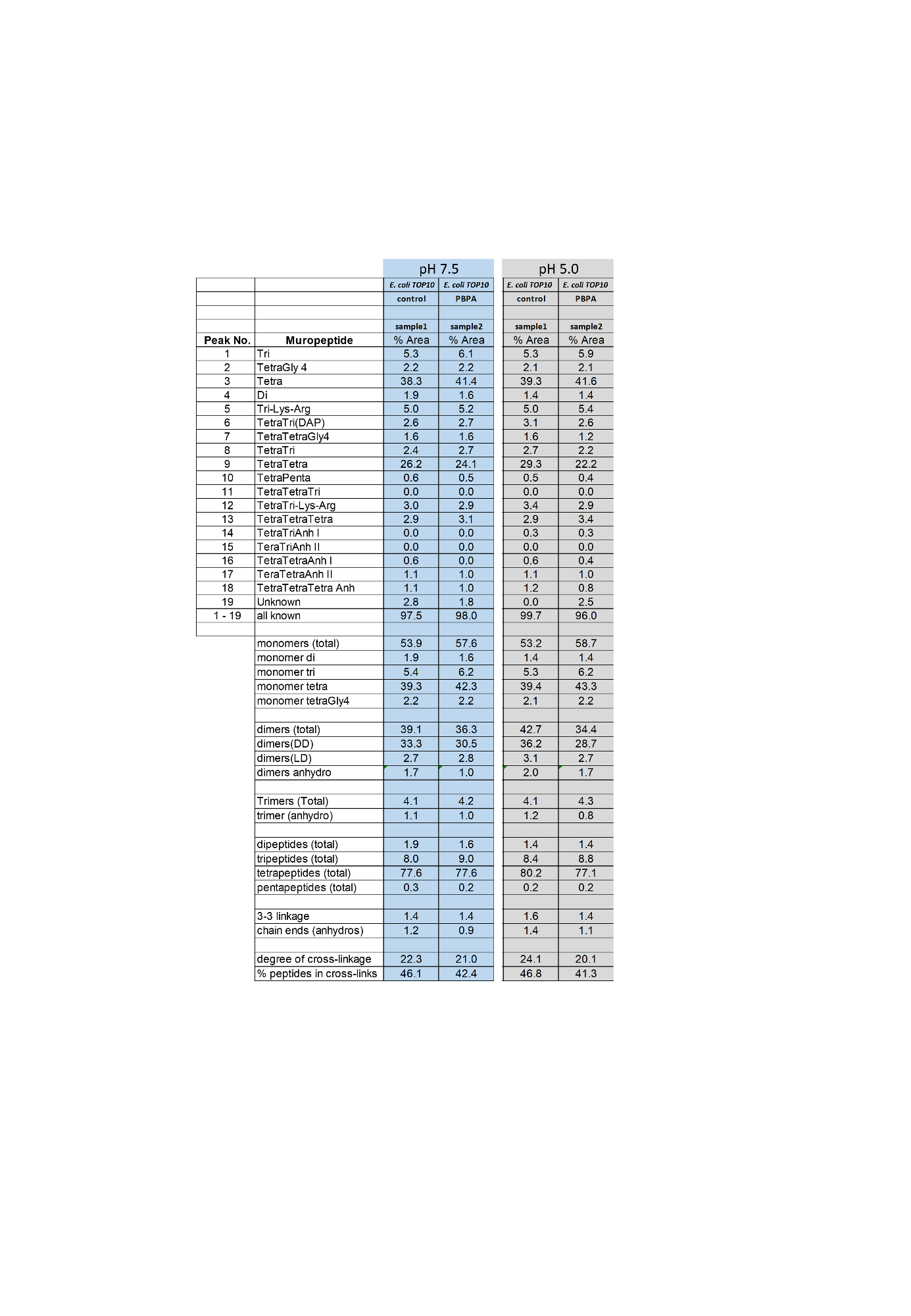


**Supplementary Table S4.** Relative abundance (%) of muropeptides detected in PG isolated from *E. coli* TOP10 (control), *E. coli TOP10/rhaA::PBP-A-wt* and *E. coli TOP10/pBAD43_PBP-A-wt.*


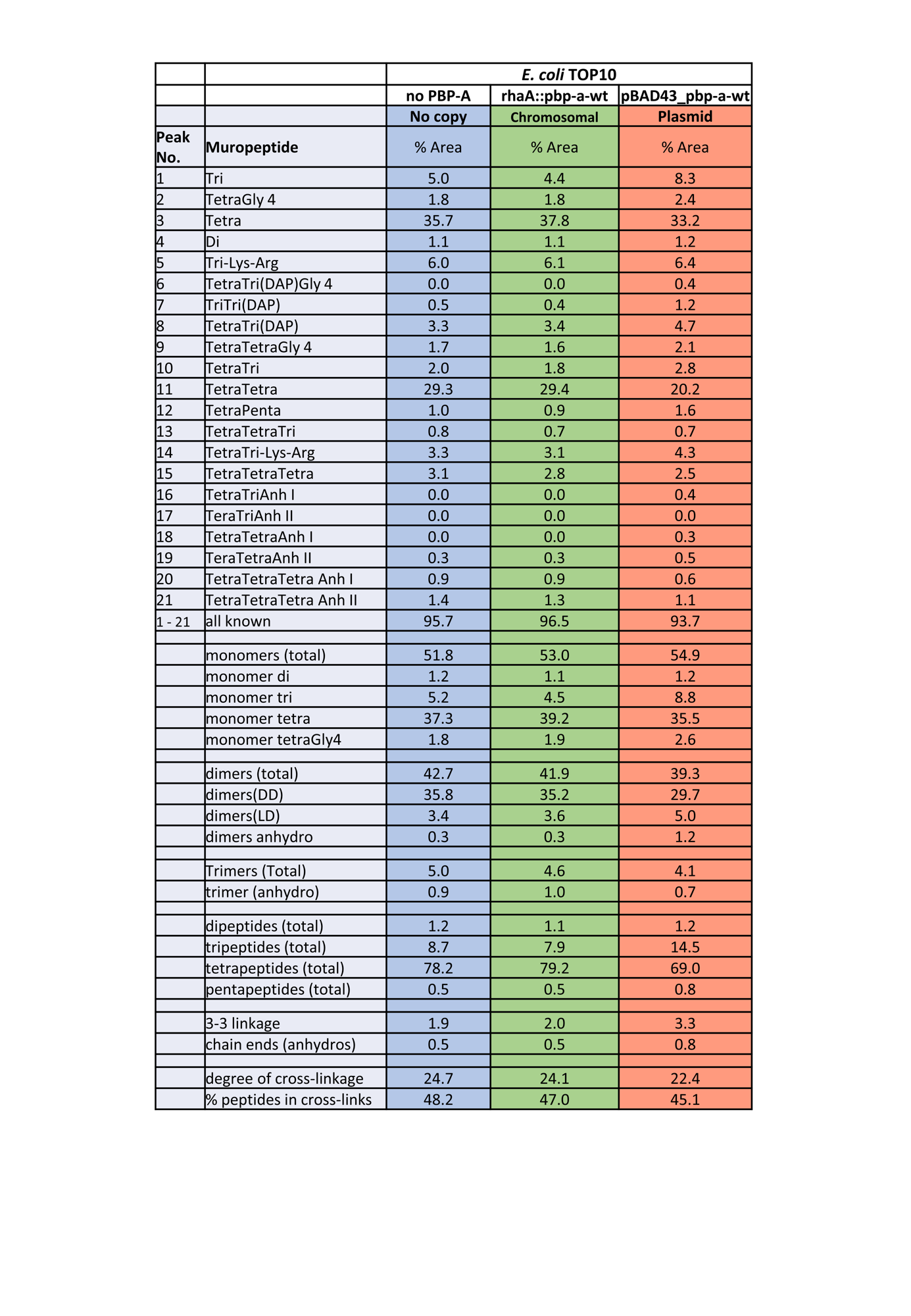


**Supplementary Table S5.** The RMSF values for the catalytic Ser61 and the tripeptide units for the PBP-A in complex with the substrate in amidated, carboxylic (COOH), and carboxylated (COO^-^) form.

| Residue  model | Ser61 | D-Ala | mDAP | isoD-Gln |
| --- | --- | --- | --- | --- |
| Amidated model | |  |  |  |
| Lowest energy | 0.3925 | 0.5331 | 2.3632 | 3.7427 |
| Lowest distance_x1 | 0.3811 | 0.5130 | 1.2802 | 1.5595 |
| Lowest distance_x2 | 0.3789 | 0.4694 | 0.9782 | 1.2238 |
| Lowest distance_x3 | 0.3830 | 0.4916 | 1.1587 | 1.6614 |
| 2nd lowest energy | 0.4027 | 0.6954 | 2.5925 | 4.0431 |
| 2^nd^ lowest distance | 4.6358 | 0.4748 | 0.9707 | 4.2648 |
| Carboxylic acid (COOH) model | |  |  | **isoD-GluH** |
| Lowest energy | 0.3811 | 0.5476 | 1.6104 | 1.9524 |
| Lowest distance | 0.3958 | 0.5362 | 2.1287 | 3.3381 |
| Lowest energy 2 | 0.4054 | 0.6136 | 2.0935 | 2.9801 |
| Lowest distance 2 | 0.3767 | 0.5319 | 1.9990 | 3.6126 |
| Carboxylated (COO-) model | |  |  | **isoD-Glu** |
| Lowest energy | 0.3574 | 0.4565 | 0.5429 | 1.0957 |
| Lowest distance | 0.3757 | 0.5101 | 0.4594 | 0.5419 |
| Lowest energy 2 | 0.4246 | 1.0679 | 1.6327 | 2.6708 |
| Lowest distance 2 | 0.6325 | 1.2643 | 2.4779 | 2.3573 |
|  |  |  |  |  |

**Supplementary Table S6**. Clustering summary for the systems bearing amidated, carboxylic and carboxylated tripeptides. Each system/repetition contains equal number of frames (250000 frames/each).

| **Combined amidated tripeptide models** | | |  |  |  |  |
| --- | --- | --- | --- | --- | --- | --- |
| **#Cluster** | **Frames** | **Frac** | **AvgDist** | **Stdev** | **Centroid** | **AvgCDist** |
| 0 | 743998 | 0.992 | 1.042 | 0.137 | 490922 | 0.839 |
| 1 | 3292 | 0.004 | 0.819 | 0.078 | 139362 | 1.18 |
| 2 | 748 | 0.001 | 0 | 0 | 145999 | 1.466 |
| 3 | 506 | 0.001 | 0 | 0 | 246427 | 1.316 |
| 4 | 432 | 0.001 | 0 | 0 | 567891 | 1.347 |
| 5 | 317 | 0 | 0 | 0 | 151517 | 1.369 |
| 6 | 260 | 0 | 0 | 0 | 165698 | 1.201 |
| 7 | 178 | 0 | 0 | 0 | 61739 | 1.344 |
| 8 | 153 | 0 | 0 | 0 | 586960 | 1.407 |
| 9 | 116 | 0 | 0 | 0 | 125909 | 1.249 |
| **Combined carboxylic tripeptide models** | | |  |  |  |  |
| **#Cluster** | **Frames** | **Frac** | **AvgDist** | **Stdev** | **Centroid** | **AvgCDist** |
| 0 | 983027 | 0.983 | 1.289 | 0.202 | 484271 | 0.879 |
| 1 | 6206 | 0.006 | 0 | 0 | 134995 | 1.577 |
| 2 | 3929 | 0.004 | 0 | 0 | 760362 | 1.485 |
| 3 | 1998 | 0.002 | 0 | 0 | 662100 | 1.599 |
| 4 | 1947 | 0.002 | 0 | 0 | 618070 | 1.464 |
| 5 | 1463 | 0.001 | 0 | 0 | 290768 | 1.457 |
| 6 | 669 | 0.001 | 0 | 0 | 617456 | 1.527 |
| 7 | 338 | 0 | 0 | 0 | 218025 | 1.393 |
| 8 | 272 | 0 | 0 | 0 | 865042 | 1.562 |
| 9 | 151 | 0 | 0 | 0 | 127131 | 1.662 |
| **Combined carboxylated tripeptide models** | | |  |  |  |  |
| **#Cluster** | **Frames** | **Frac** | **AvgDist** | **Stdev** | **Centroid** | **AvgCDist** |
| 0 | 955487 | 0.955 | 1.416 | 0.277 | 804784 | 0.958 |
| 1 | 23909 | 0.024 | 0 | 0 | 444771 | 1.984 |
| 2 | 8031 | 0.008 | 0 | 0 | 610242 | 1.617 |
| 3 | 5502 | 0.006 | 0.773 | 0.088 | 733996 | 1.776 |
| 4 | 2763 | 0.003 | 0 | 0 | 690358 | 1.914 |
| 5 | 2135 | 0.002 | 0.863 | 0.163 | 738128 | 1.751 |
| 6 | 1027 | 0.001 | 0.884 | 0.059 | 4865 | 1.667 |
| 7 | 535 | 0.001 | 0 | 0 | 5269 | 1.618 |
| 8 | 382 | 0 | 0 | 0 | 674608 | 2.025 |
| 9 | 229 | 0 | 0.472 | 0 | 2528 | 1.743 |

**Supplementary Table S7**. List of the models used for MD simulations.

| **Model’s feature** | **Interface energy** | **Distance [Å] and angle [°]** |
| --- | --- | --- |
| **Amidated model** |  |  |
| Lowest energy | -14.984 | 2.76, 117.03 |
| 2^nd^ lowest energy | -14.4668 | 3.05, 109.99 |
| Lowest distance | -12.627 | 2.68, 100.08 |
| 2^nd^ lowest distance | -11.4369 | 2.70, 119.23 |
| **Carboxylic acid (COOH) model** |  |  |
| Lowest energy | -13.347 | 3.03, 100.64 |
| 2^nd^ lowest energy | -11.0543 | 3.17, 128.89 |
| Lowest distance | -12.0838 | 2.92, 105.40 |
| 2^nd^ lowest distance | -10.9536 | 3.30, 129.59 |
| **Carboxylate (COO-) model** |  |  |
| Lowest energy | -16.6621 | 2.69, 119.11 |
| 2^nd^ lowest energy | -12.4651 | 2.90, 101.19 |
| Lowest distance | -11.7382 | 2.65, 109.56 |
| 2^nd^ lowest distance | -10.8301 | 2.74, 103.50 |

**Supplementary Table S8.** Summary of the initial setup.

| Initial Structure | Water | ions | Total atoms | Simulation time | Initial simulation box size |
| --- | --- | --- | --- | --- | --- |
| Amidated tripeptide model | | | | | |
| lowest energy | 15957 | 1 Cl- | 52052 | 1 µs | 90.7405064 x 90.7405064 x 90.7405064 |
| lowest distance | 16198 | 1 Cl- | 52775 | 3 x 1 µs | 91.2361944 x 91.2361944 x 91.2361944 |
| lowest energy 2 | 16371 | 1 Cl- | 53294 | 1 µs | 91.5695637 x 91.5695637 x 91.5695637 |
| lowest distance 2 | 15750 | 1 Cl- | 51431 | 1 µs | 90.4217046 x 90.4217046 x 90.4217046 |
|  | | | **Total** | **6 µs** |  |
| Carboxylic tripeptide (COOH) model | | | | | |
| lowest energy | 15887 | 1 Cl- | 51841 | 1 µs | 90.6493609 x 90.6493609 x 90.6493609 |
| lowest distance | 15849 | 1 Cl- | 51727 | 1 µs | 90.5718746 x 90.5718746 x 90.5718746 |
| lowest energy 2 | 15875 | 1 Cl- | 51805 | 1 µs | 90.6458994 x 90.6458994 x 90.6458994 |
| lowest distance 2 | 15886 | 1 Cl- | 51838 | 1 µs | 90.6461160 x 90.6461160 x 90.6461160 |
|  | | | **Total** | **4 µs** |  |
| Carboxylated tripeptide (COO^-^) model | | | | | |
| lowest energy | 15209 | none | 49805 | 1 µs | 89.5866996 x 89.5866996 x 89.5866996 |
| lowest distance | 15963 | none | 52067 | 1 µs | 90.8091934 x 90.8091934 x 90.8091934 |
| lowest energy 2 | 16148 | none | 52622 | 1 µs | 91.0954884 x 91.0954884 x 91.0954884 |
| lowest distance 2 | 15493 | none | 50657 | 1 µs | 90.0351914 x 90.0351914 x 90.0351914 |
| Angles: 109.4712190, 109.4712190, 109.4712190 | | | **Total** | **4 µs** |  |
|  | | | **Grand Total** | **14 µs** |  |

**Supplementary Table S9**. Prepin files for the parameterized tripeptide units.

ISG, ISC, ISO, and DAP refer D-iGln, D-iGluH, D-iGlu and meso-diaminopimelic acid, respectively.

**D-iGln**


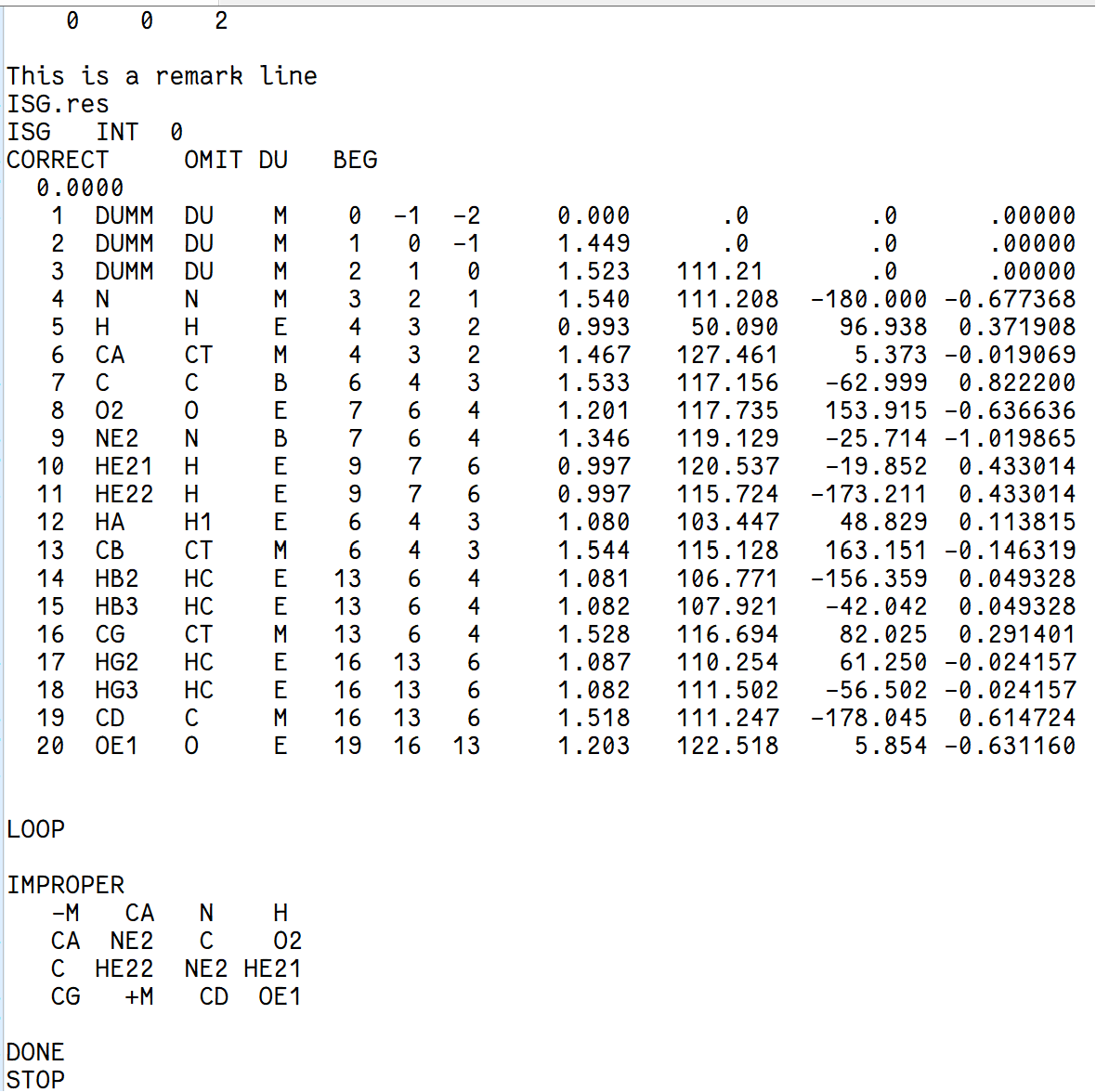


**mDAP**


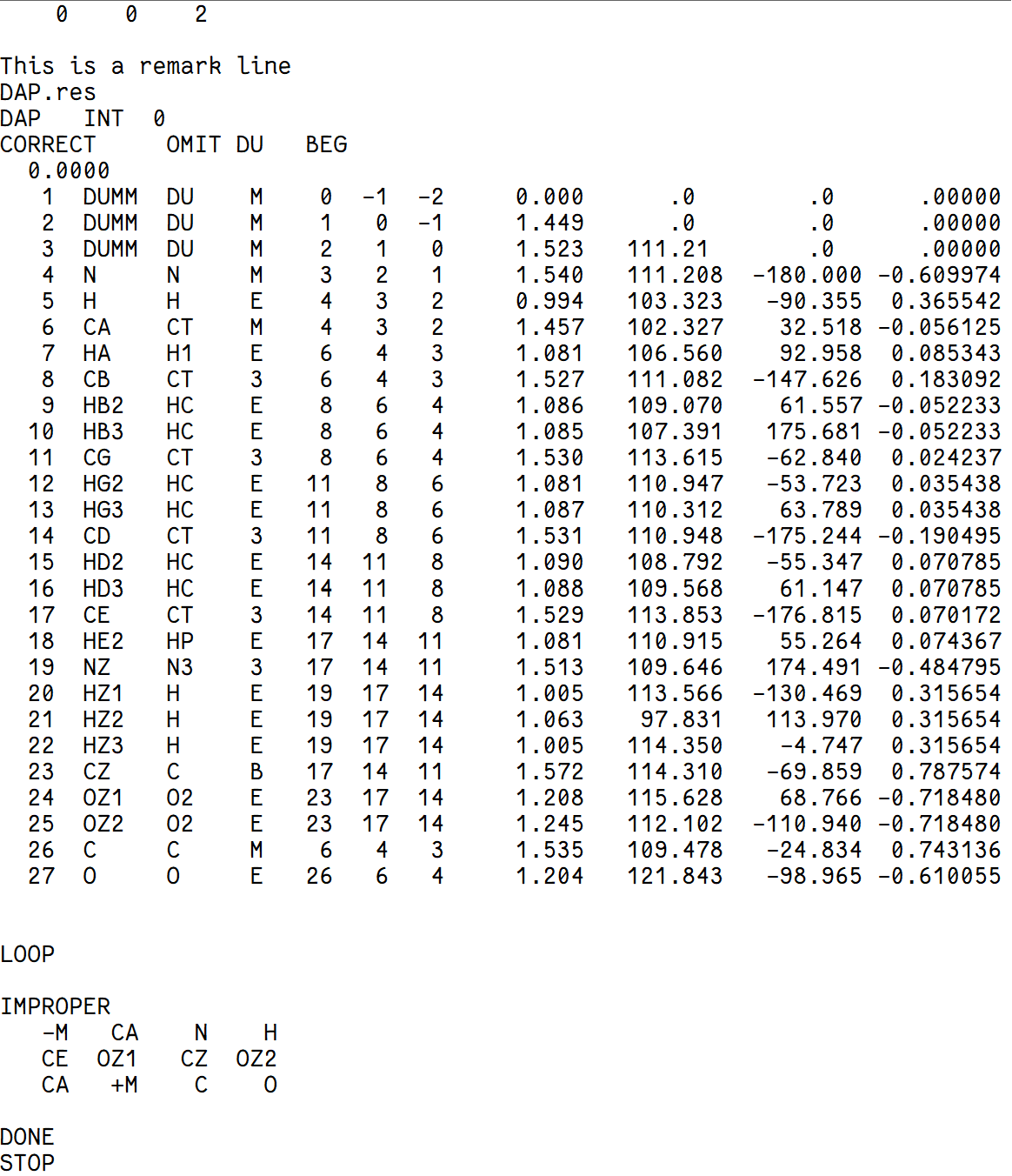


**SAC (Ser + D-Ala)**

**
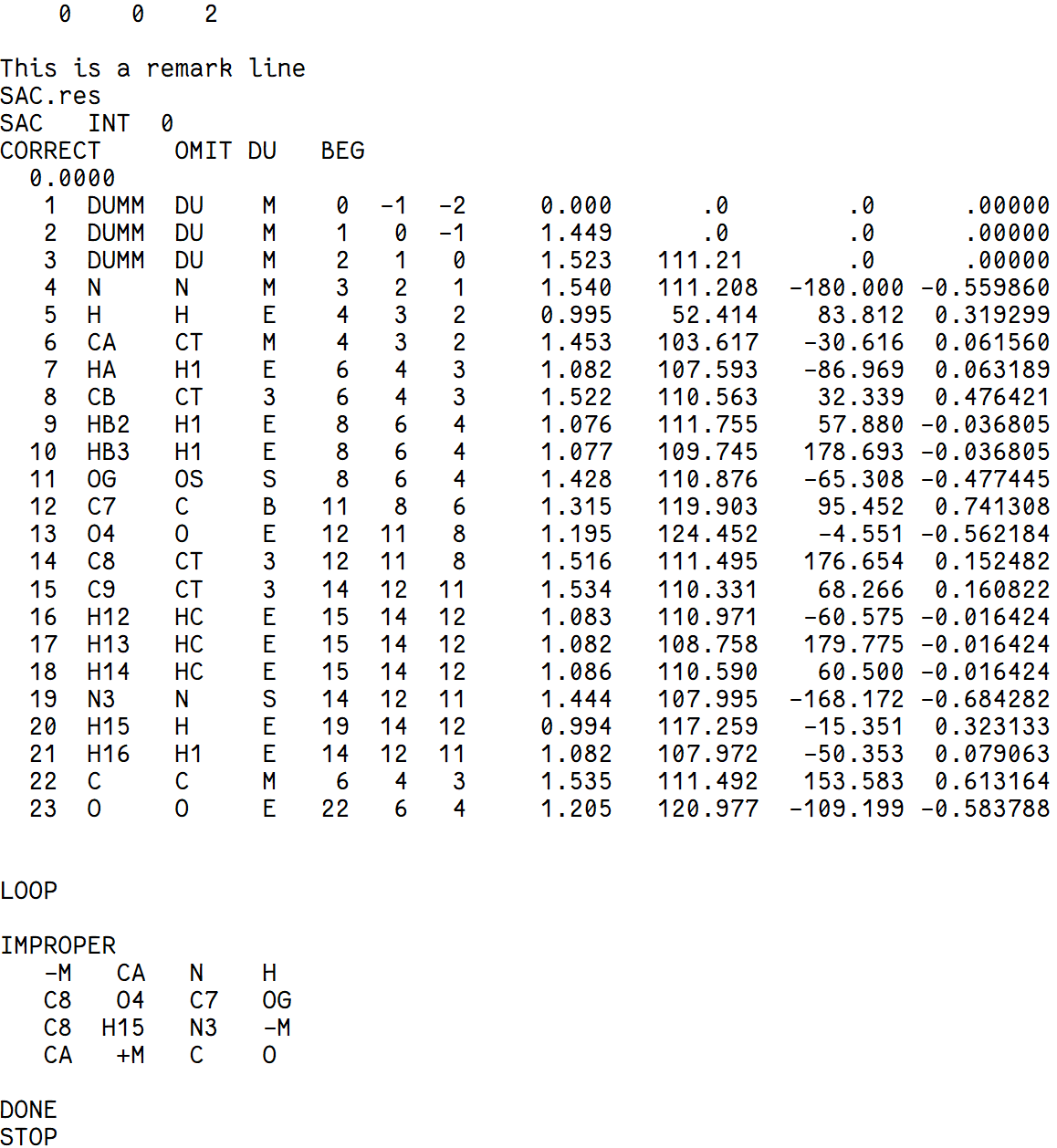
**

**D-iGluH**

**
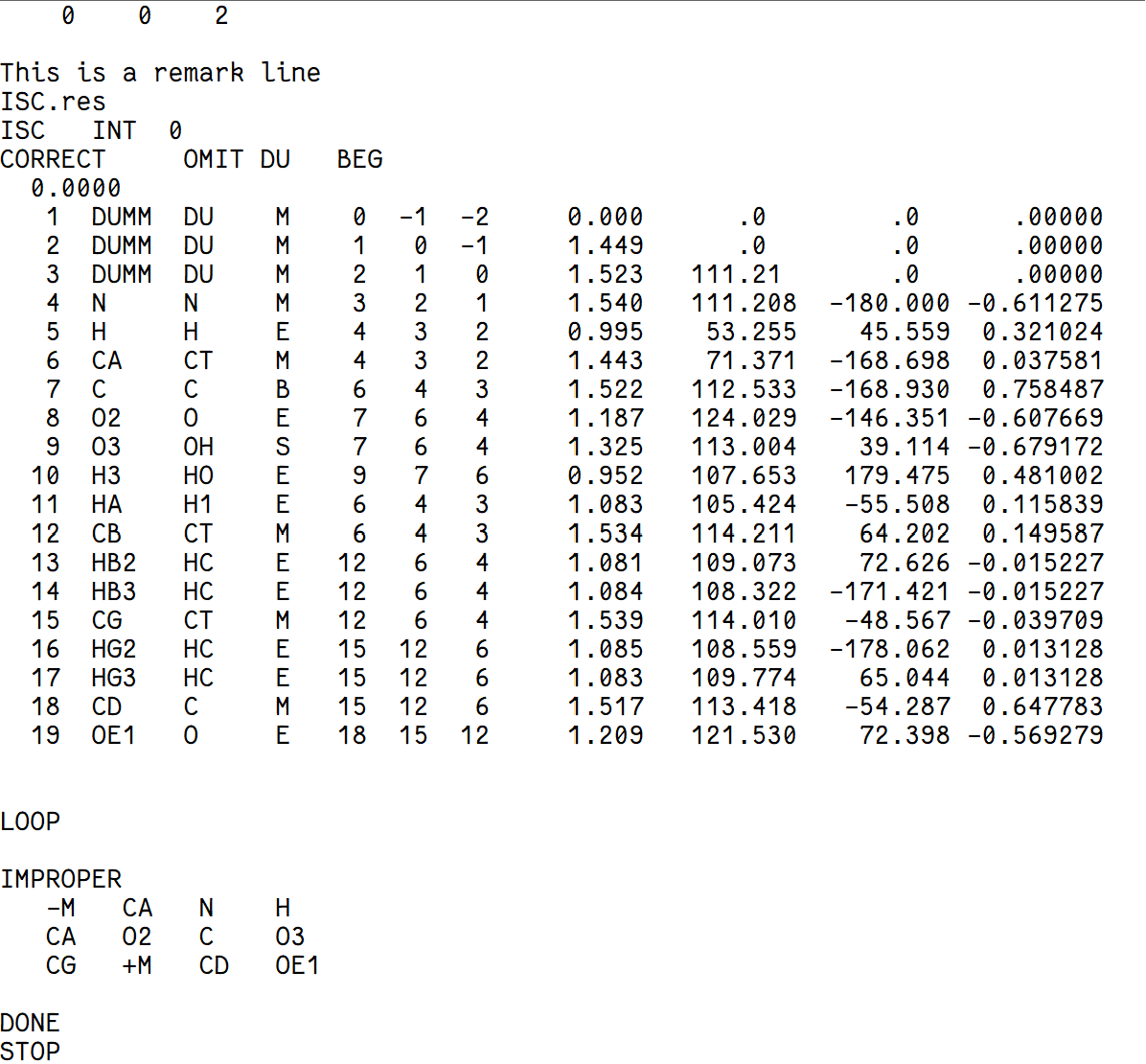
**

**D-iGlu**

**
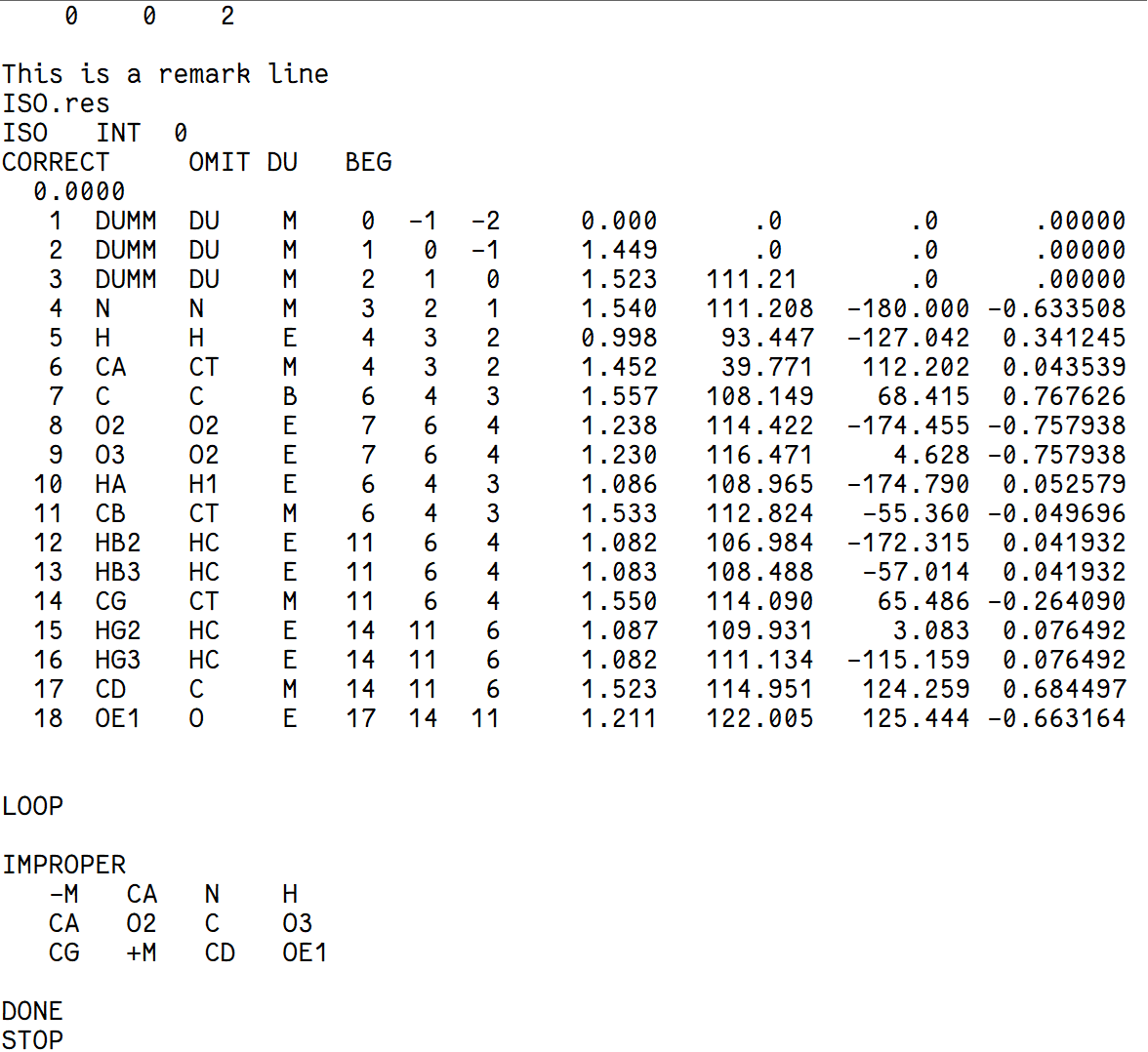
**

**Supplementary Figure S1.** Capillary electrophoresis analysis of PBP-A assay with amidated pentapeptide. During time-dependent capillary electrophoresis, 2 peaks were showing increase in peak area and 1 peak was showing decrease, while other peaks remained constant. Among the increasing peaks, peak of D-alanine (D-Ala) was verified by adding D-Ala in a positive control assay, hence the other increasing peak would be the Tetrapeptide (Tetra) product.


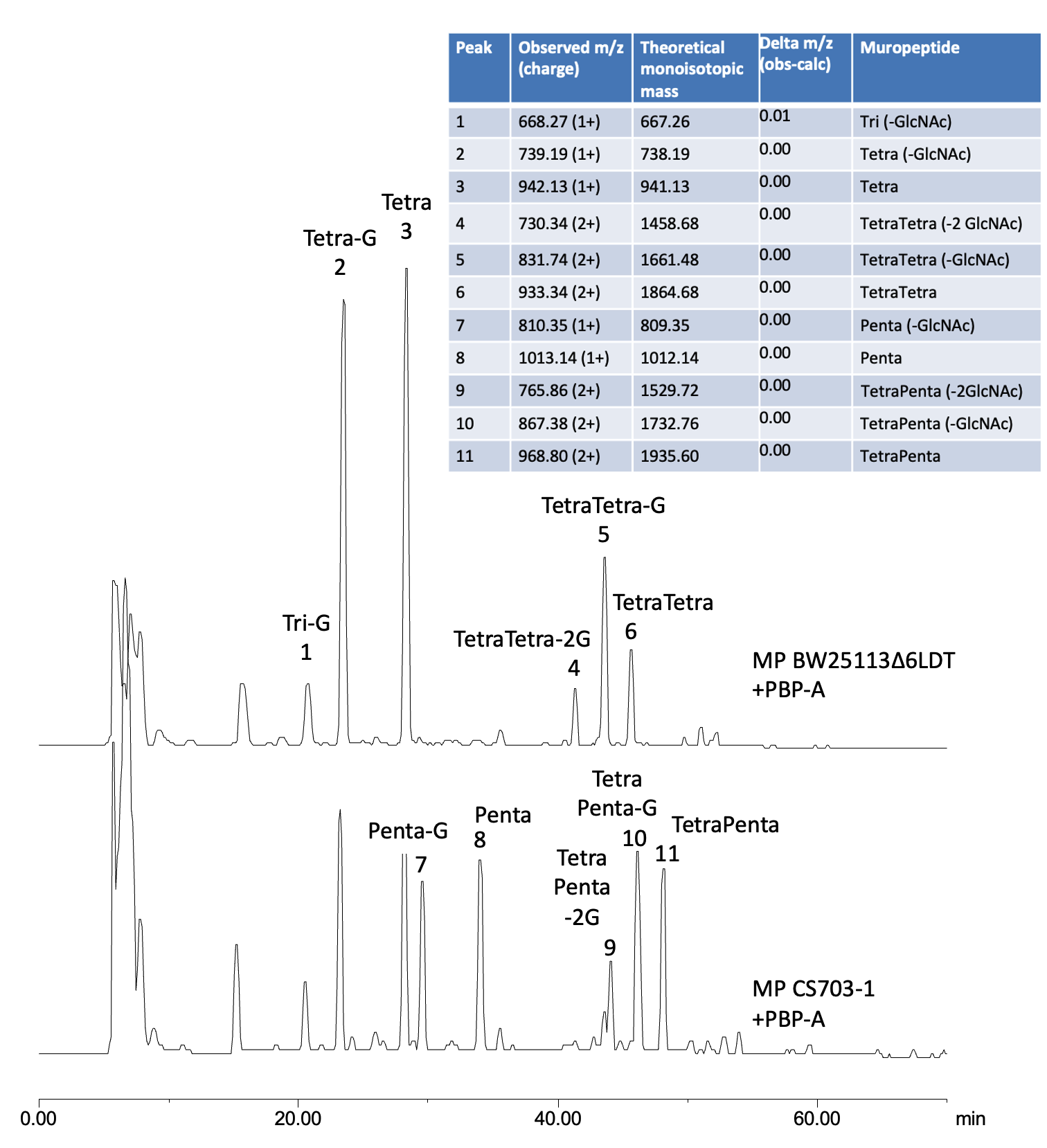


**Supplementary Figure S2.** HPLC peak detection in assay of PBP-As with purified PG. The table on the right represent MS analysis of labelled peaks identified in HPLC


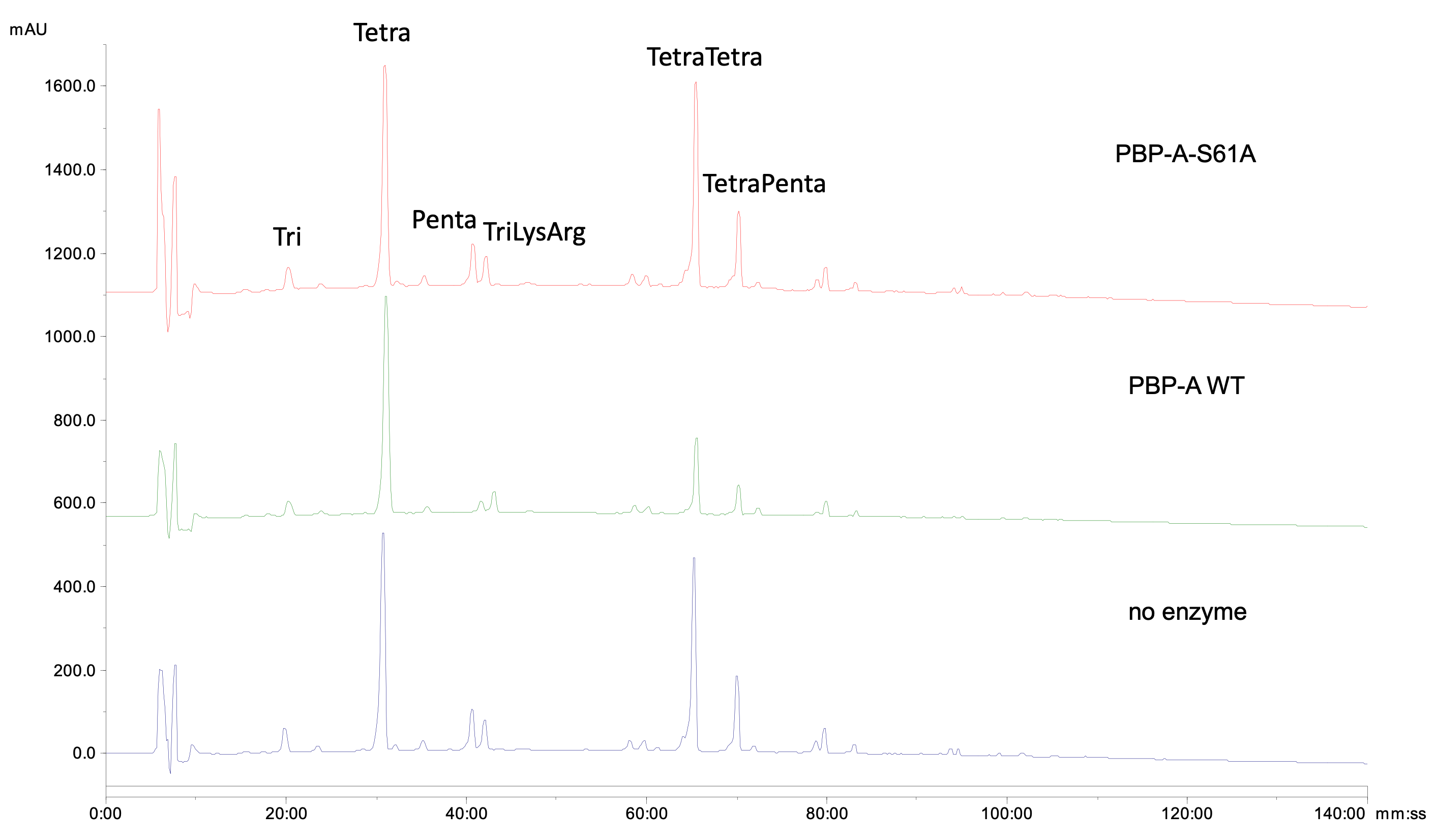


**Supplementary Figure S3.** Assaying PBP-A wt and catalytic mutant PBP-A-S61A on muropeptides from *E. coli*. For these experiments, the PBP-A enzymes were purified from a cytoplasmic expression system.


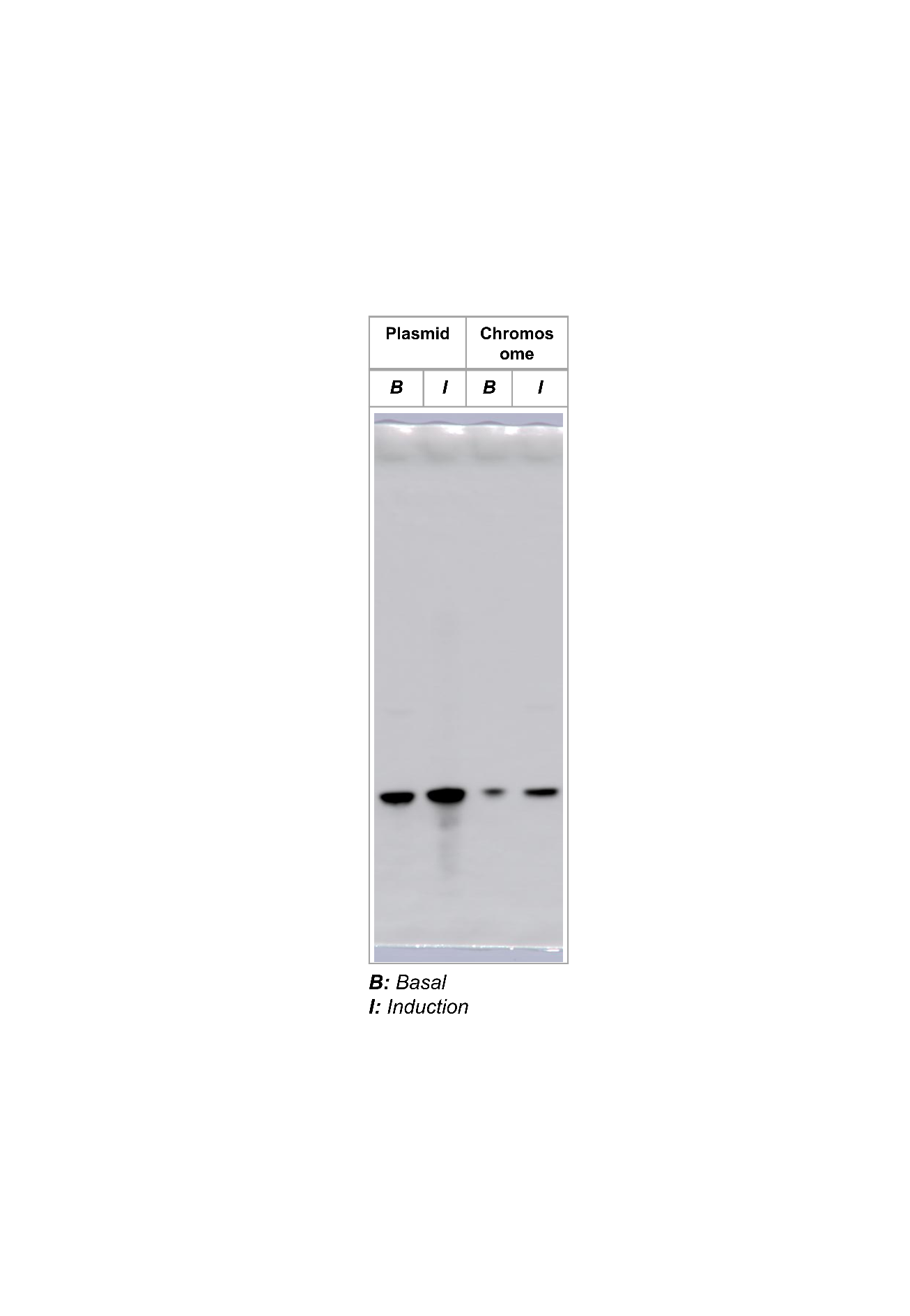


**Supplementary Figure S4.** Effect of copy number on expression level of PBP-A evaluated by western blot analysis of periplasmic extracts of *E. coli* expressing PBP-A under low-copy plasmid (pBAD43) and chromosomal context. Equal amounts of cells were subjected to periplasmic extraction.


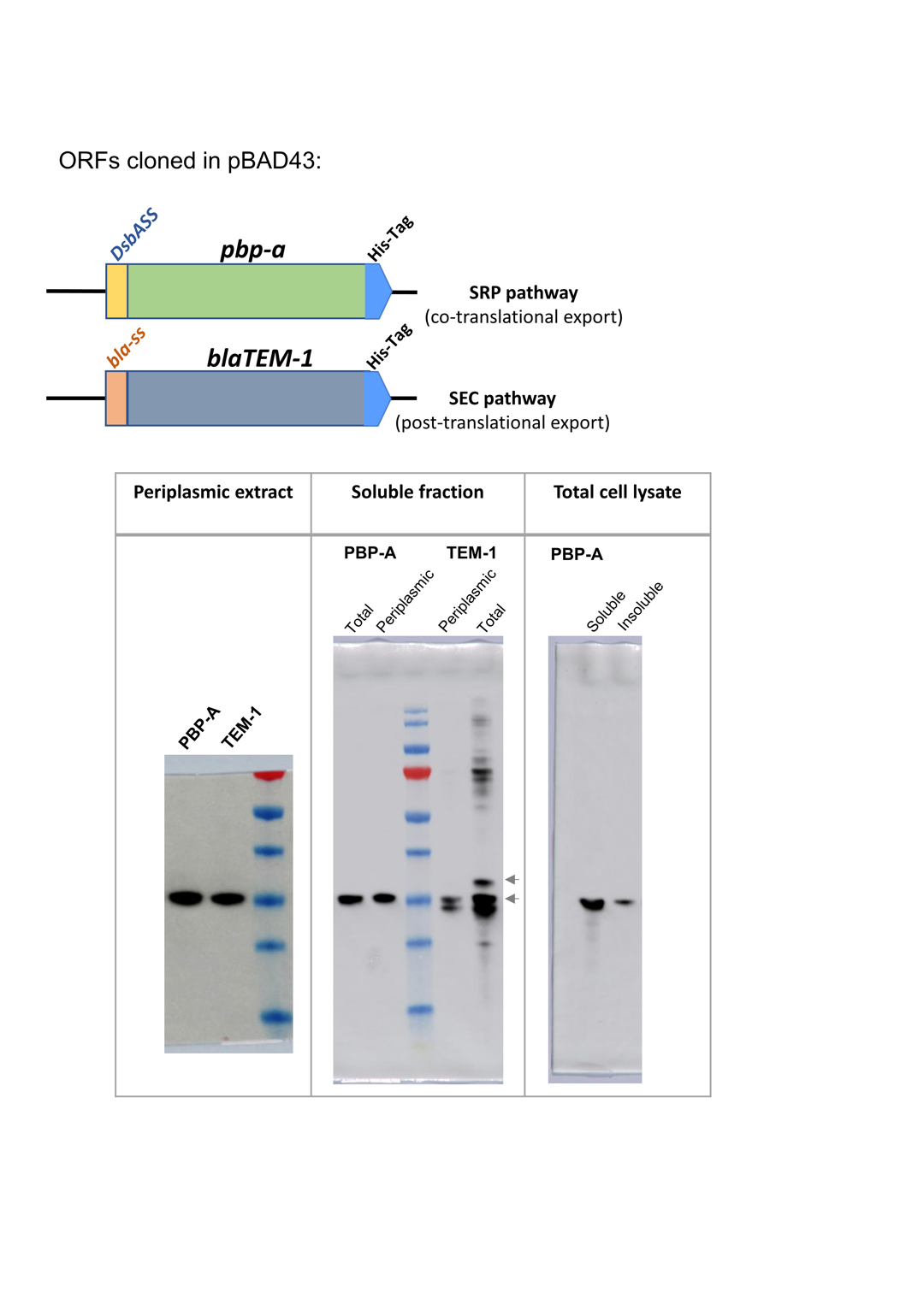


**Supplementary Figure S5.** Translocation of proteins into periplasm of *E. coli*. Upper left: the genetic constructs of *pbp-A* and bla genes are shown in schematic, where a DsbA signal sequence (DsbAss) is fused to N-termini of *pbp-A* gene to facilitate a co-translational export while the TEM-1 gene is fused to its native β-lactamase signal sequence (bla-ss). Lower left: western blot of soluble fractions from periplasmic and total cell lysate of *E. coli* confirms the co-translational expression of mature PBP-A proteins without any pre-mature intermediates, while for a post-translationally mediated TEM-1 protein shows presence of both mature (30.5 kDa) and pre-mature or un-exported (33.0 kDa) proteins. Right: Western blotting of total soluble and insoluble fractions from total cell lysate of *E. coli*/pBAD43_DsbAss-PBP-As confirms that PBP-A almost entirely exist in soluble form.


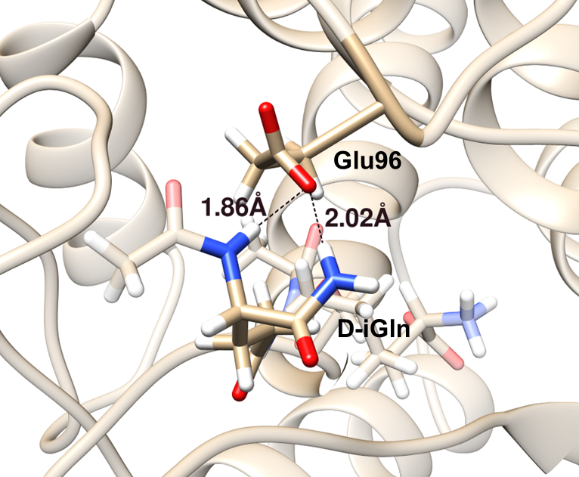


**Supplementary Figure S6.** Representation of key H-bond interactions between D-iGln and Glu96 (representative structure from cluster C0).


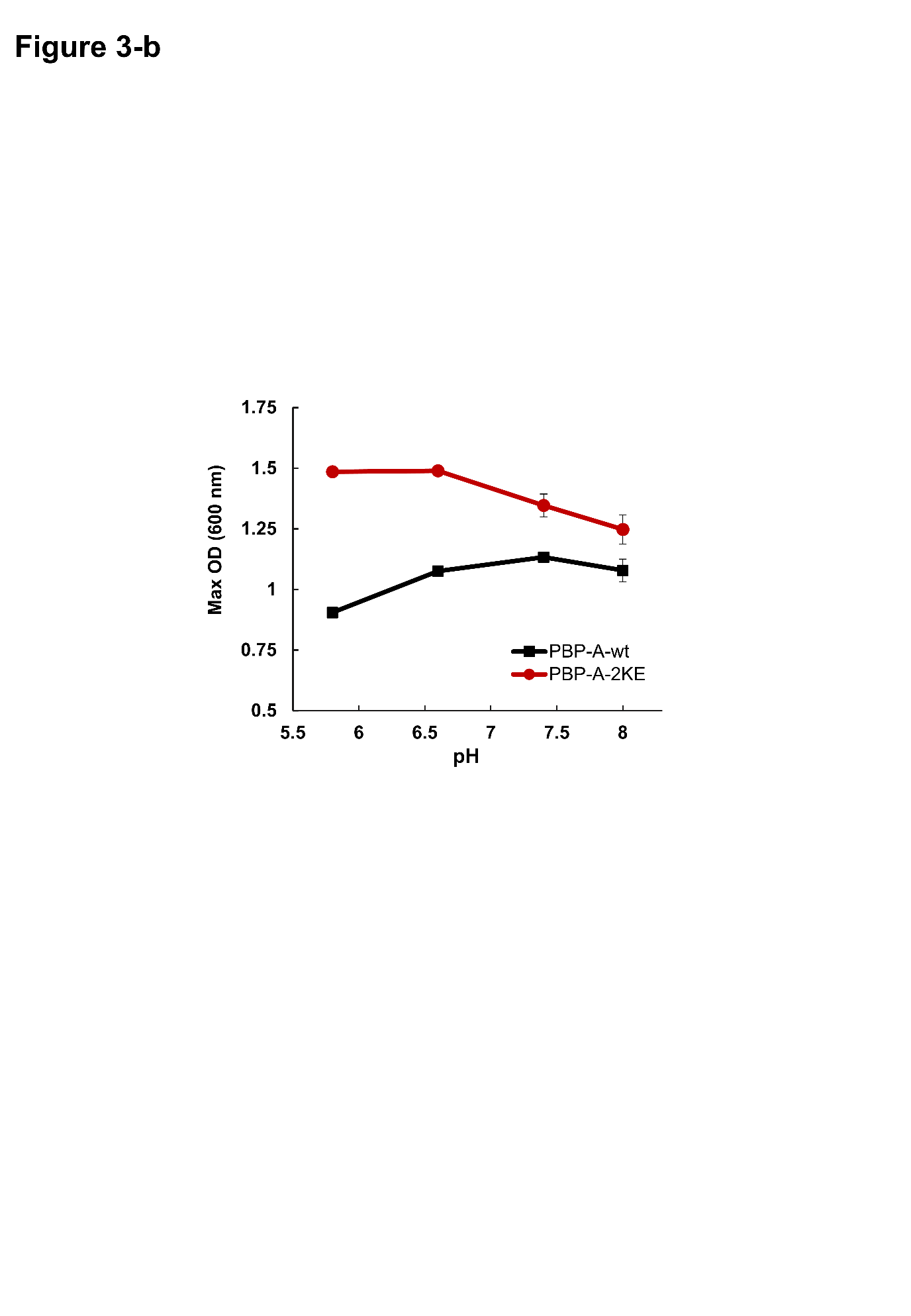


**Supplementary Figure S7.** Maximum population density (max OD_600_) of *E. coli* cultures at different pH. Comparison of pH profiles between *E. coli* TOP10 cells expressing PBP-A-wt (pI 7.56), and PBP-A-2KE harboring two substitutions of surface lysines K104 and K212 into glutamates (Glu, E) (pI 5.99). Theoretical isoelectric point of proteins were calculated using *ExPASy Server (*[*https://web.expasy.org/compute_pi/*](https://web.expasy.org/compute_pi/)*)*.

**
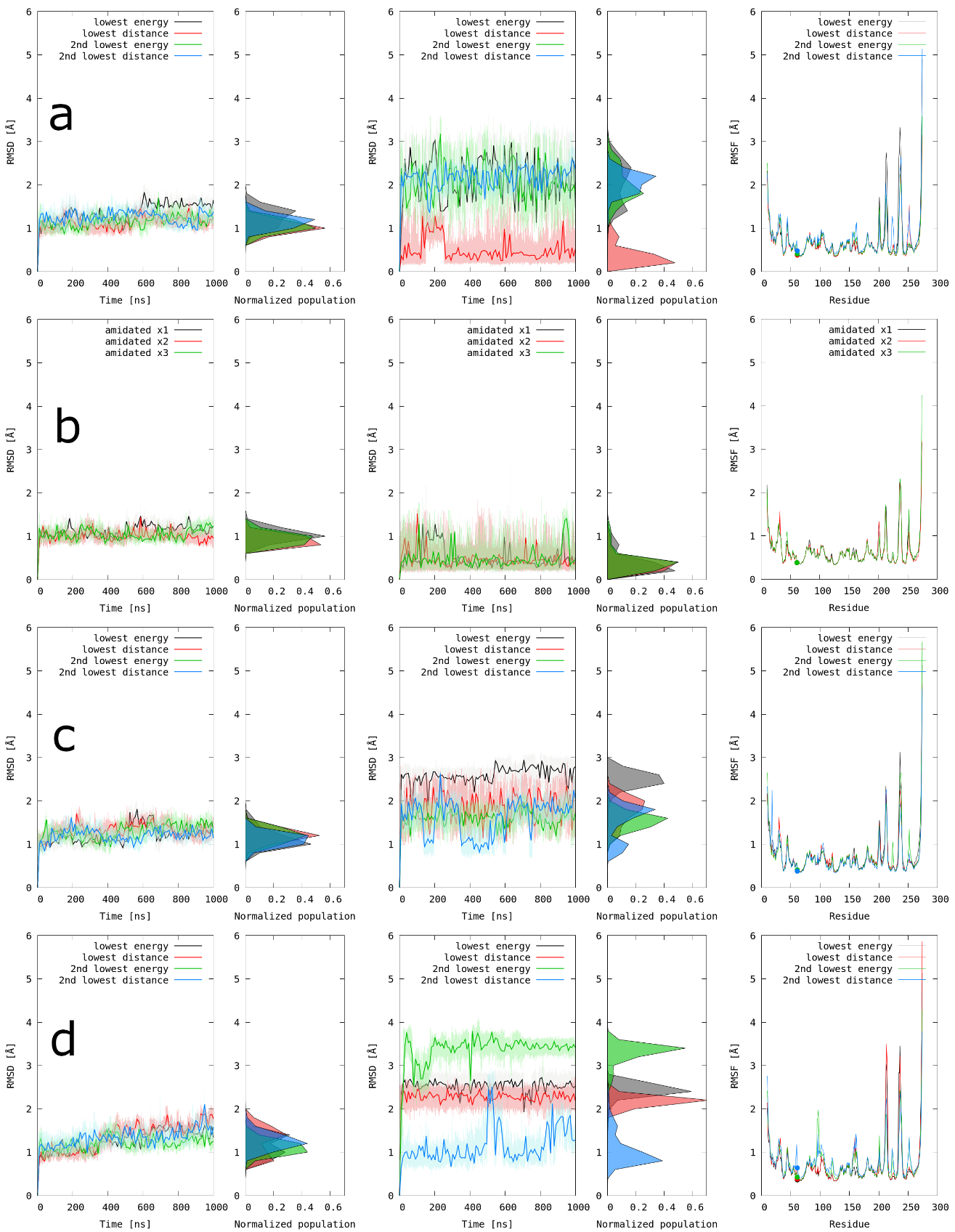
**

**Supplementary Figure S8**. Backbone RMSD for the protein (right panel) and tripeptide (middle panel) with respect to first structures after equilibration for PBP-A in complex with (a) amidated, (b) amidated conformer with the lowest distance, (c) carboxylic (COOH), and (d) carboxylated (COO^-^) tripeptides. In the RMSF plot (left panel) the active site Ser61 marked as a dot.

**Distance analysis for the three repetitions of the amidated lowest distance conformer**


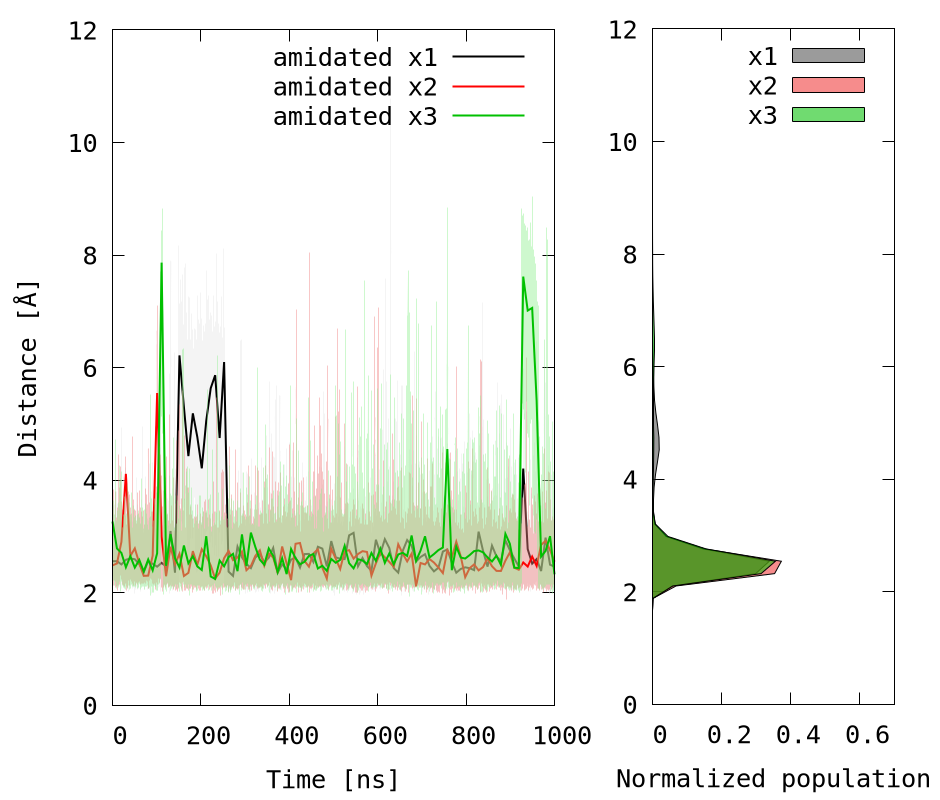

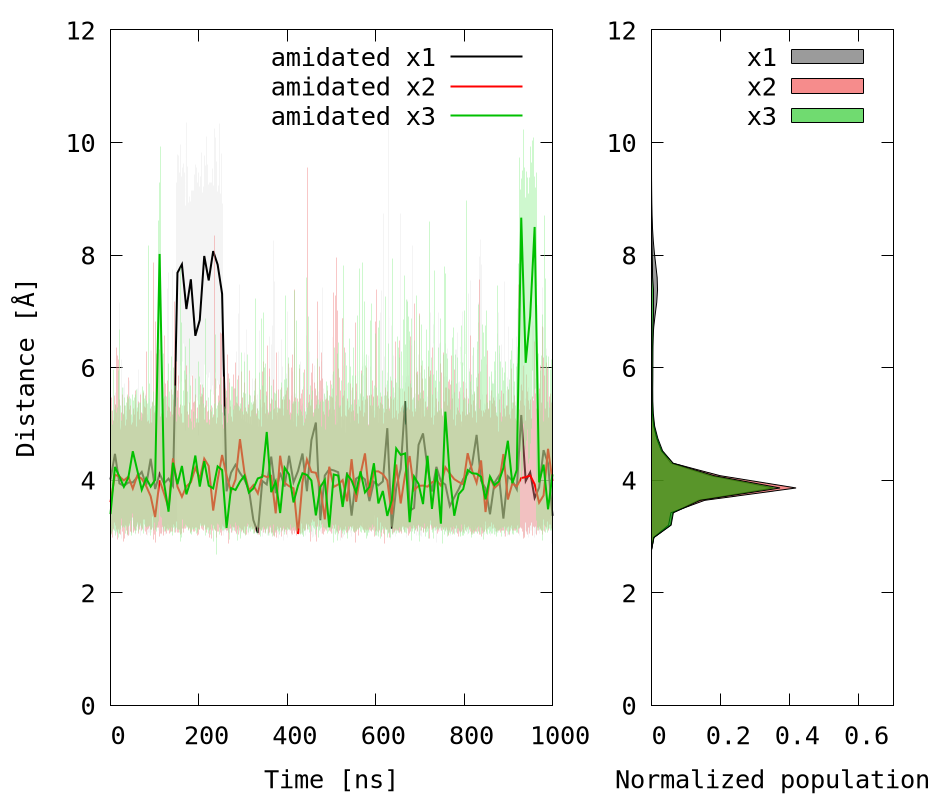


**Supplementary Figure S9.** Distance plot between (a) D-iGln@NE2 - Glu96@CD and (b) D-iGln@H - Glu96@CD followed by corresponding histograms (c) and (d).


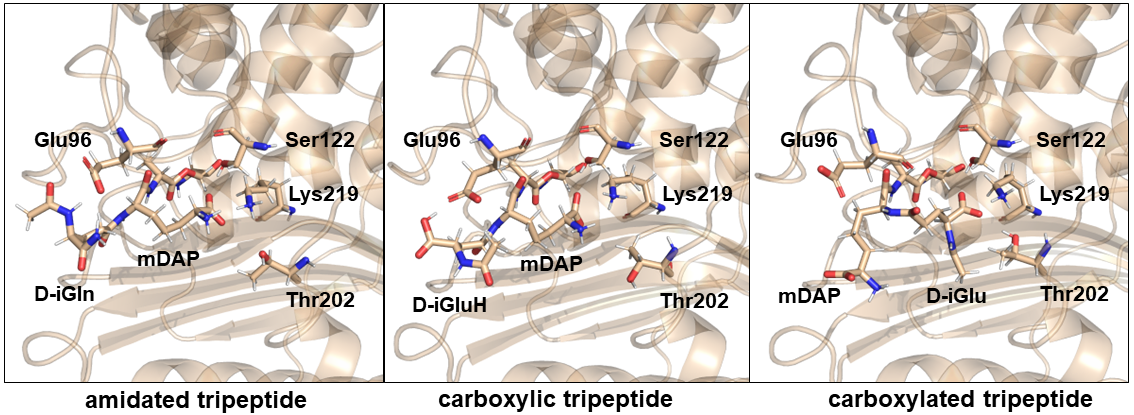


**Supplementary Figure S10**. Representation of the tripeptide orientations for each model (representative structure from cluster C0 for each system).
